# Quantification of the cellular dose and characterization of nanoparticle transport during in vitro testing

**DOI:** 10.1186/s12989-016-0157-1

**Published:** 2016-08-24

**Authors:** Grigore Rischitor, Mariantonietta Parracino, Rita La Spina, Patrizia Urbán, Isaac Ojea-Jiménez, Elena Bellido, Andrea Valsesia, Sabrina Gioria, Robin Capomaccio, Agnieszka Kinsner-Ovaskainen, Douglas Gilliland, François Rossi, Pascal Colpo

**Affiliations:** 1 European Commission Joint Research Centre, Institute for Health and Consumer and Protection, Nanobiosciences Unit, Via E. Fermi 2749, 21027 Ispra, VA Italy; 2 Present address : Nanoimmunotech S.L., Av. de la Autonomía, 50003 Zaragoza, Spain

**Keywords:** Nanoparticles, Cell uptake, Colloidal stability, Nanoparticle characterization, Nanoparticle transport, Dosimetry, *In vitro* assay

## Abstract

**Background:**

The constant increase of the use of nanomaterials in consumer products is making increasingly urgent that standardized and reliable in vitro test methods for toxicity screening be made available to the scientific community. For this purpose, the determination of the cellular dose, i.e. the amount of nanomaterials effectively in contact with the cells is fundamental for a trustworthy determination of nanomaterial dose responses. This has often been overlooked in the literature making it difficult to undertake a comparison of datasets from different studies. Characterization of the mechanisms involved in nanomaterial transport and the determination of the cellular dose is essential for the development of predictive numerical models and reliable in vitro screening methods.

**Results:**

This work aims to relate key physico-chemical properties of gold nanoparticles (NPs) to the kinetics of their deposition on the cellular monolayer. Firstly, an extensive characterization of NPs in complete culture cell medium was performed to determine the diameter and the apparent mass density of the formed NP-serum protein complexes. Subsequently, the kinetics of deposition were studied by UV*-vis* absorbance measurements in the presence or absence of cells. The fraction of NPs deposited on the cellular layer was found to be highly dependent on NP size and apparent density because these two parameters influence the NP transport. The NP deposition occurred in two phases: phase 1, which consists of cellular uptake driven by the NP-cell affinity, and phase 2 consisting mainly of NP deposition onto the cellular membrane.

**Conclusion:**

The fraction of deposited NPs is very different from the initial concentration applied in the in vitro assay, and is highly dependent of the size and density of the NPs, on the associated transport rate and on the exposure duration. This study shows that an accurate characterization is needed and suitable experimental conditions such as initial concentration of NPs and liquid height in the wells has to be considered since they strongly influence the cellular dose and the nature of interactions of NPs with the cells.

**Electronic supplementary material:**

The online version of this article (doi:10.1186/s12989-016-0157-1) contains supplementary material, which is available to authorized users.

## Background

Nanotechnology has the potential to create new classes of materials and devices that can have a huge impact on many domains ranging from cosmetics, agriculture, and food to health applications. Consequently, a large increase in the development and use of new nanomaterials (NMs) is expected in the near future [1]. To enable a quick and reliable NM safety assessment there is an urgent need for robust, standardized and reliable test methods for toxicity screening.

In vitro test methods are essential tool for mechanistic toxicity studies in basic research, for toxicity screening purposes, and for regulatory testing. However, the development and optimization of in vitro methods for NM hazard assessment is not straightforward. The large diversity of NMs makes the evaluation of their interactions with biological systems (i.e. cells and proteins) very complex and time consuming. The variety of NM types with different physico/chemical properties (size, shape, chemical composition, surface chemistries) used in consumer products raises specific challenges especially in term of sample preparation and potential interferences with the in vitro assay that can affect the assay outcome [2]. Moreover, the properties of the tested NMs can change significantly during the tests further complicating the data interpretation. In the last decade, a great deal of effort has been applied to the development of in vitro methods for NMs. Nevertheless, the large number of parameters and the complexity of the phenomena involved place question marks over the consistency of some of the studies performed until now [3–5]. For instance, the characterization of the protein corona formed around the NMs when in contact with serum proteins is essential since it has an essential role in the NM interactions with cells [6–9]. In particular, it has been shown that biomolecules secreted by the cells can modify the biological identity of the protein corona thus influencing the NM agglomeration state and cell membrane affinity [8]. A careful and extensive characterization of NMs behavior both before and during toxicity assessment is therefore needed in order to address these issues [4, 5].

Another factor, which is essential for the trustworthy interpretation of results from in vitro tests is the determination of the dose of NMs that are effectively in contact with the cells during the assay. The cellular dose is the crucial parameter for correctly determining the dose response to NMs, which is necessary for toxicity assessment [10, 11]. In most in vitro studies, the initial concentrations of NMs dispersed in the culture medium were considered as the effective concentrations, as it is normally done for water-soluble chemicals. Nevertheless, in contrast to chemical compounds, NMs are solid objects poorly soluble in culture medium, and that depending of their properties (size, mass density and solubility) undergo gravitational and/or diffusive transport to reach the cell monolayer [12–14]. Furthermore, dynamic modifications of the NM characteristics may occur upon mixing with biological media resulting in agglomeration, aggregation and formation of the protein corona. These alterations might change the size and apparent density of the formed NM-protein complexes. This in turn may directly influence their transport towards the cell monolayer [13]. Consequently, the number of NMs effectively reaching the cell monolayer may differ drastically from the nominal dose i.e. the initial concentration used in the assay. The importance of this issue has raised significant interest within the nanotoxicology community leading to the publication of noteworthy works addressing the modeling and the experimental aspects of dosimetry [12–20]. These references underline the importance of characterizing the interactions between the NMs and the serum proteins contained in culture media in order to determine the size and the mass density taking into consideration the presence of the protein corona or of agglomerates which are essential parameters affecting NM transport toward the cell monolayer. As a result, most recent publications have started to pay closer attention to these aspects in order to increase the relevance of the data produced.

The determination of the NM cellular dose is not trivial. It is complementary to cellular uptake studies since the kinetics of cellular uptake is governed by two mechanisms i.e. the transport of the NMs to the cell monolayer and their effective internalization [21, 22]. The cellular dose, i.e. the sum of the NMs taken up by the cells and bound to the cell membranes depends both on the transport of NMs and on the NM-cell membrane affinity. Both are closely related since the local NM concentration and transport in a culture well is the result of the equilibrium between diffusion, sedimentation and the uptake rate (affinity) of the NMs by the cells [18]. Cellular uptake has been widely studied for different types of NPs. For instance, the determination of gold NPs taken-up by the cells can be performed by mass spectrometry [22, 23] and fluorescently labeled silica NPs by flow cytometry [24]. These studies overall show that, for a given cell system, the uptake depends on the NM size and surface chemical properties.

In the present work, we describe a method combining the exhaustive characterization of the NPs and the determination of the cellular dose based on a previously described method using UV-*vis* absorbance measurements [14, 25] to relate the physico-chemical properties of gold NPs to the kinetics of deposition on the cellular monolayer during long-term exposure. The outstanding physical properties of gold NPs are very attractive for biomedical applications for instance for diagnostics in vivo and targeted delivery of drugs [26]. Because of their biocompatibility, relative stability and inertness, and easiness of preparation, gold NPs have been used as model in many studies of NP-cell interactions [22, 26, 27] in order to understand the mechanisms of uptake and their fate after internalisation. In our study, commercial and in-house synthesized NPs with sizes ranging from 10 nm to 80 nm have been used in order to cover the two modes of transport: diffusion and gravitational sedimentation. Characterization of NPs in complete culture medium was performed by Dynamic Light Scattering (DLS) and Centrifugal Liquid Sedimentation (CLS) to determine the stability, size and apparent density of the NPs. UV*-vis* spectroscopy technique has been used to study the kinetics of NP deposition in cell culture medium alone and in the presence of A549 lung epithelial cells. The experiments have been performed for 72 h to determine the time evolution of the NP deposition on the cell monolayer.

## Results

### Nanoparticle characterization

Prior to studying their transport toward the cell monolayer in 96 well plate, the NPs were characterized, firstly, in water and then in cell culture medium (CCM) to assess their stability in the cell culture conditions and to determine their size and apparent mass density resulting from their interactions with serum proteins.

Two different sets of NPs were used in this study: commercial and in house synthetized gold NPs. The first set of NPs (CO20, CO40, CO80) were surfactant stabilized (supplier proprietary surfactant used as stabilizer) whereas in house synthetized NPs (HM15, HM35 and HM75) were citrate-stabilized (see Methods for more details).

Firstly, Transmission Electron Microscopy (TEM) analyses were performed in order to measure the core size of the NPs. TEM images of both sets of NPs are shown in Additional file 1: Figures S1a, b and the corresponding nominal sizes are presented in Table 1. The results of image analysis show that both types of NPs have a mono-disperse core size distribution and comparable size range.

**Table 1:** Summary of TEM, CLS and DLS measurements performed in water. D_core_: Diameter measured by TEM, *D*_*core*_^*calc*^ calculated value of D_core_ corresponding to a core-shell structure of apparent density ρ_app_ composed of a core of density 19.3 g.cm^-3^ and a shell of density 1.064 g.cm^-3^ (nominal density of the CLS gradient)

	TEM		DLS			CLS			
	D_core_ (nm)	*D*_*core*_^*calc*^ (nm)	D_h_ (nm)	PdI_h_	Z-pot mV	D_hs_ (nm)	HHW	PdI_hs_	ρ_app_ g/cm^3^
HM15	11.3 ± 1.8	8.69	20.3	0.04	−37.0	7.8	1.5	1.08	3.8
HM35	29.6 ± 4.7	31.9	34.6	0.14	−40.3	29.7	4.5	1.05	13.6
HM75	68.9 ± 14.4	65.4	71.8	0.24	−44.6	62.5	11.9	1.09	14.8
CO20	15.7 ± 3.2	21.3	32.9	0.11	−16.1	17.1	3.0	2.34	6.0
CO40	35.5 ± 5.5	39.8	54.4	0.12	−20.2	38.3	11.0	1.17	8.2
CO80	72.8 ± 8.1	81.3	95.4	0.07	−25.1	75.1	15.0	1.21	12.4

*D*_*h*_ : average hydrodynamic diameter, *PdI*_*h*_ : poly-dispersion index measured by DLS_,_ *Z-Pot*: Zeta potential, D_hs:_ hydrodynamic diameter measured by CLS calculated with a density of 19.3 g.cm^−3^, *HHW:* half height width of the measured size distribution, PdI_hs_: poly-dispersion index measure by CLS, ρ_app_: apparent density calculated with the method described in [31])

DLS and CLS measurements were initially performed in water and the measured sizes are reported in Table 1. The hydrodynamic diameters measured by DLS in water show the different behavior of the HM and CO sets of NPs. Whereas each pair of nanoparticles (HM15/CO20, HM35/CO40, and HM75/CO80) has the same core size range as measured by TEM, their respective sizes are very different when measured in water suspension. Compared to the core sizes measured by TEM, the measured hydrodynamic diameters (by DLS and CLS) of the CO series of the NPs were significantly larger (≈10 nm) than those measured of the HM series (≈ 5 nm).

Measurements of the Zeta potential of the NPs reported in Table 1 show that the CO series of NPs have a less negative surface charge than the in-house synthetized NPs. The hydrophilicity of surfactant stabilizer of the commercial NPs and the citrate stabilizer of the in-house synthetized NPs has been assessed by contact angle measurements. Briefly, both solvents in which the NPs are dispersed were spotted on a plain gold surface. Contact angle was measured and was found to be lower than 11 degrees for both stabilizing agents showing their high hydrophilic character.

CLS is known to be one of the most accurate techniques to evaluate the size of NPs in dispersion [28–30]. This method is based on the measurement of the sedimentation time of the NPs across density gradient during centrifugation. The time of sedimentation is a function of the fixed experimental parameters such as viscosity of the fluid, density of the gradient of sucrose, the rotational speed and the dimension of the rotating disk and two unknown elements related to the NP properties, namely the NP mass density and hydrodynamic diameter. The CLS hydrodynamic diameter can be calculated from the sedimentation time with the following equation:

1 $$ {D}_{hs}=\sqrt{\frac{ln\frac{Rf}{R0}\times 18\eta }{t\ \left({\rho}_{NP}-{\rho}_f\right){\omega}^2}} $$

*D*_*hs*_ and *ρ*_*NP*_ are the diameter and the mass density of the NPs respectively. *t* is the sedimentation time, *ρ*_*f*_ density of the sucrose gradient. *R*_*f*_ and *R*_*o*_ are external radii of the rotating disk, *η* the fluid viscosity, and *ω* the angular velocity of the rotor.

Generally, NP diameters are calculated from the CLS measurements by using the mass density of the bulk material. This assumption overestimates the real apparent density of the NPs dispersed in water since it does not account for the contribution of the hydration layer present around of the NPs. The use of the relation (1) with the mass density of the bulk material therefore leads to an underestimation of the calculated hydrodynamic diameter. The hydrodynamic diameters calculated with this assumption are presented in Table 1 and the corresponding spectra presented in Additional file 1: Figures S2a and S2b. As expected in this condition, the values of hydrodynamic diameter calculated by CLS are lower than those obtained by DLS. Overall, the shape of CLS curves and the corresponding poly-dispersity index confirm that the NPs are mono-disperse as observed by TEM.

Because it is an essential element for the NP transport characterization the apparent mass density of the NPs, taking into account the presence of the hydration layer, has been determined by combining DLS and CLS techniques [31]. This method is based on the use of the hydrodynamic diameter of the NPs measured by DLS in the CLS Eq. (1) (D_hs_ = D_h_) to calculate apparent NPs complex density (ρ_NP_ = ρ_app_).

The apparent densities calculated by this method are presented in Table 1. As expected, the results show that the calculated apparent densities are lower than the gold bulk density (19.3 g · cm^−3^) since they take into account the density of the NPs in the presence of the hydration layer and of the surfactant stabilizer presence for the commercial NPs. The apparent densities obtained for HM15, HM35 and HM75 are in a good agreement with measurements performed by analytical ultra-centrifuge for citrate stabilized NPs with similar diameters [32].

The validity of the approach can be verified by calculating the size of the NP core with the previously calculated mass density. The mass of the complex can be expressed as the sum of the mass of the core and the mass of the hydration shell:

2 $$ {\rho}_{app}{V}_h={\rho}_{core}{V}_{core}+{\rho}_{shell}{V}_{shell} $$

where ρ_shell_ is the density of the hydrated layer, including the protein corona if present, V_h_, V_core_ and V_shell_ respectively the volume corresponding to the DLS hydrodynamic diameter, to the core diameters and shell thickness. The diameter of the core of the NPs can be calculated by using the relation:

3 $$ {D}_{core}^{calc}={D}_{h\kern0.5em }{\left(\frac{\rho_{app}-{\rho}_{shell}}{\rho_{core}-{\rho}_{shell}}\right)}^{\frac{1}{3}} $$

The *D*_*core*_^*calc*^ values calculated for value of ρ_shell_ of 1.064 g · cm^−3^ (nominal CLS sucrose gradient mass density), which represents the density of the hydration layer are presented in Table 1. Since the gold NPs have a rather high mass density, the values of *D*_*core*_^*calc*^ do not vary significantly with the value of ρ_shell_ and follow linearly the values of D_core_ measured by TEM with a linear correlation coefficient of 0.98.

Subsequently, the NPs have been characterized in CCM by DLS, CLS and UV-*vis* spectrometry techniques (Tables 2 and 3). We observed that the HM15 NPs immediately agglomerate upon contact with the cell culture medium. Agglomeration has been observed in both DLS and CLS measurements. This poor stability of the HM15 NPs makes them unsuitable for the purpose of this study.

**Table 2:** NP characterisation in complete cell culture medium conditions

	DLS in CCM		CPS in CCM			
	D_h_ nm	PdI	Sedimention time (s) in Water	Sedimention time (s) in CCM	ρ_app_ g.cm^−3^	*D*_*core*_^*calc*^ (nm)
HM35	69.7	0.29	28.9	42.1	3.30	34.5
HM75	101.9	0.16	7.7	10.3	6.21	66.6
CO20	35.8	0.11	97.5	138.0	4.32	19.8
CO40	66.5	0.16	25.0	31.5	5.00	39.8
CO80	116.7	0.09	5.3	7.1	6.72	78.9

**Table 3:** UV*-vis* SPR peak values measured in water and in CCM

Sample	HM35	HM75	CO20	CO 40	CO80
Water	527 nm	544 nm	523 nm	530 nm	555 nm
CCM	537 nm	552 nm	524 nm	532 nm	556 nm

In cell culture conditions, the NPs are coated by serum proteins forming NP-protein complexes. As expected, the diameters of NP-protein complexes measured by DLS in CCM increased as compared to diameters measured in water due to the formation of the protein corona surrounding the NPs. The intensity based size distribution profiles measured by DLS presented in Additional file 1: Figures S3a and S3b show an increase in NP diameters due to the presence of the protein corona, while the shapes of the NPs size distributions are similar to those measured in water demonstrating that no agglomeration occurred. The measured diameter in CCM is slightly higher for the citrate stabilized NPs (HM series) than for the surfactant stabilized NPs (CO series) in relation to the influence of the surface chemistry on protein-NP interactions. In order to assess the influence of the protein corona on the sedimentation time, the CLS measurements in water and in CCM have been compared. The curves of the UV-*vis* absorbance measured at the CLS detector location versus time are presented in Additional file 1: Figures S4a and S4b.

The CLS sedimentation time significantly increases for all NPs in presence of proteins in cell culture medium. This shift toward higher sedimentation times is a result of the increase of NP hydrodynamic diameters and the decrease of the NP apparent densities due the protein corona.

The NPs hydrodynamic diameters measured by DLS have been used to calculate the apparent density of the complex NP-proteins (Table 2). As expected, the results show a further decrease of the apparent density due to the presence of the protein corona as compared to those obtained in water.

Here again, the values of *D*_*core*_^*calc*^ reported in Table 2, for values of ρ_shell_ of 1.125 g.cm^-3^ [26] correlate linearly with the values obtained by TEM (Table 1) with a correlation coefficient of 0.98.

UV*-vis* spectra of the NPs in the same conditions have been measured (Additional file 1: Figure S5a and b) and the obtained maximum surface plasmon resonance (SPR) peaks are arranged in Table 3.

A red shift of the SPR peak position for HM35 and HM75 NPs is observed because of the protein corona formation. Noticeably, only a very slight shift is observed with the CO20, CO40 and CO80 NPs. This shift is smaller than the one observed with the HM nanoparticles. A possible reason is that, due to the presence of the surface layer, proteins remain too far from the NPs surface, therefore do not affect significantly the SPR signal.

In order to confirm that the increase of diameter observed by the different measurement techniques is due to the presence of the protein corona, Sodium Dodecyl Sulphate Polyacrylamide gel electrophoresis (SDS Page) analysis has been performed. The NPs were incubated with cell culture medium containing serum proteins for 72 h. The SDS PAGE gel images are presented in Additional file 1: Figure S6 for two time points (t = 0 and 72 h). The images show clear protein bands with significant changes in corona composition between the two series of NPs. We can observe the appearance of a band at 91 kDa for the CO NP series which is absent for the HM series of NPs. For both type of NPs an increase in band intensity is observed when the incubation time increase from t =0 to 72 h.

In order to assess qualitatively if secreted biomolecules by the cells have an influence on the biological identity of the protein corona, SDS analysis has been performed by using CCM conditioned with A549 cell for 72 h (Additional file 1: Figure S7). Again the SDS PAGE gel images show an important difference in protein corona composition between the commercial and in house synthetized NPs.

### Cell monolayer integrity assessment

Cell monolayer integrity is an essential prerequisite for the determination of the cellular dose over 72 h. Briefly, A549 cells were cultured in 96 well plates until 100 % confluence was reached. Bright field image of the cell monolayer culture are shown in Additional file 1: Figure S8a. Cell monolayers then were exposed to different sizes and types of gold NPs at final concentrations of 40 and 60 μM for respectively HM and CO sets of NPs for up to 72 h (11 time points were studied). At the end of exposure time, the cells were stained with Hoechst 33342 and Propidium Iodide (Additional file 1: Figure S8b). Hoechst 33342 is staining nuclei of both healthy and dead cells. Only necrotic cells are permeable to propidium iodide, so dead cells were identified by their intense red fluorescence. Data acquisition and analysis was performed as described in Materials and Methods. The ratio of living cells/dead cells of the exposed A549 cells was analyzed with the IN Cell Analyzer 2200 in triplicates for each of the eleven time points. Results for all time points are presented in Additional file 1: Figure S8c. Data obtained show that the cell monolayer is not affected by the exposure to gold NPs at all conditions tested. Cell viability was found above 95 % in respect to the control cells for all treatments and up to 72 h exposure always, indicating that the cell monolayer integrity was not affected by treatments of both types of NPs. The number of cells was constant over all the different experiment durations and conditions.

### UV*-vis* spectrum acquisition analysis

The method used for the determination of the effective dose reaching the cell monolayer is based on the measurement of UV-*vis* spectra of the CCM supernatant removed from the wells at different time points of the experiments [25].

**Fig. 1:**
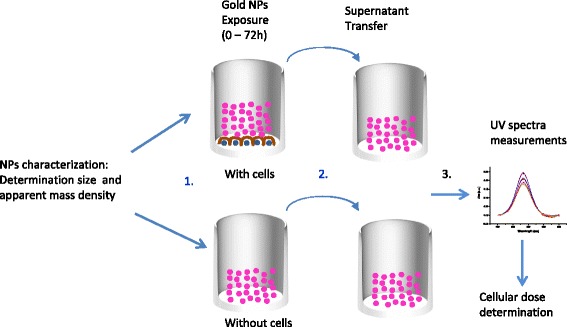
Method used to determine the effective dose reaching the cell monolayer 1) incubation of the NPs in wells with and without cells. 2) At each time point, transfer of the supernatant into an empty well for UV*-vis* measurements 3). The cellular dose is then determined by subtracting the calculated areas under the absorbance curves at t = 0 s to the one of the considered time point

**Fig. 2:**
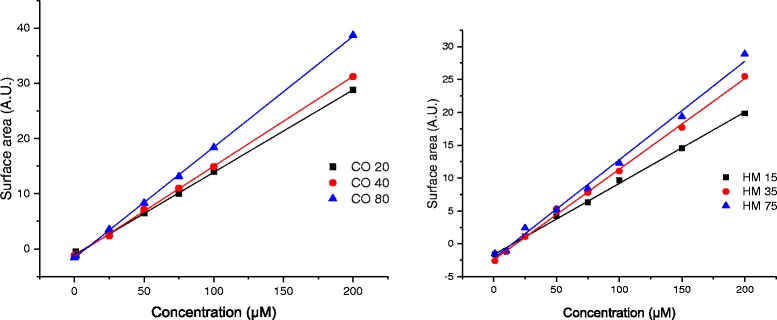
Calibration curves obtained by plotting calculated area under the UV*-vis* absorbance curves as a function of the NP concentration

The experiments have been performed in wells containing cells or only cell culture medium. The method is described in Fig. 1. In order to avoid interferences from the serum proteins, UV-*vis* measurements were performed at wavelengths between 450 nm and 650 nm (see Additional file 1: Figure S9). For a given experimental condition (liquid height and serum protein content), SPR peak intensities and positions are functions of NP concentration and agglomeration state. However, using the absolute variation of SPR peak intensities to monitor NP concentration remaining in the supernatant is complex. Indeed, the UV-*vis* spectra being measured from the bottom of the well, minor alteration in protein concentration within the sample due to cell metabolism and slight liquid evaporation during the experiments may modify and slightly shift the whole spectra intensity leading to some analysis uncertainty. In order to determine the change of concentration of the NPs presents in the supernatant at different time points, the baseline was subtracted from the average triplicate spectrum and the area under the absorbance curves was then calculated. Using this method, calibration curves versus known concentrations of gold NP were built and surface area values have been found linearly proportional to concentration of the NPs (see Fig. 2) showing the reliability of the method.

### Determination of the NP cellular dose

**Fig. 3:**
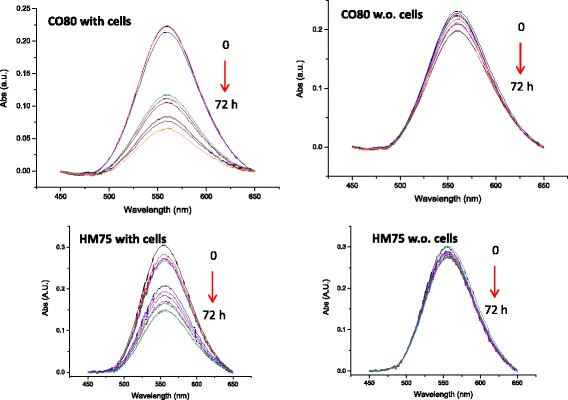
UV-*vis* spectra measured for HM75 (40 μM) with (**a**) and without cells (**b**), and for CO80 (60 μM) with (**c**) and without cells (**d**)

Measurements were performed for each time point for the five different types of gold NPs incubated in the presence or absence of cells. Raw measured UV*-vis* spectra are presented in Additional file 1: Figure S9a, b, c. Corresponding baselines subtracted spectra are presented in Fig. 3. The averages and standard deviations have been calculated for the triplicates of all measured spectra. Standard deviations were lower than 5 % of the average value for the different cases studied (raw data available upon request).

Remarkably, the variation of the area under the absorbance curves versus time is strongly influenced by the presence of the cells. Indeed, the UV*-vis* absorption peak intensities measured using supernatant taken from the wells containing the cells can be seen to decrease drastically versus time whereas only slight decreases are observed in the supernatants transferred from cell-free wells. The decreases of the UV*-vis* spectrum signals versus time are the result of the cellular uptake or the deposition of the NPs on the cell membranes or on the surface of the well. These results show that the NP-protein complexes have poor or no affinity to the polystyrene well surfaces whereas higher affinity is observed in presence of the cells. As expected, the NPs interact with the cells i.e. the NPs bind to the cell membrane and/or are internalized by the cells. The same trend has been observed for the five NPs studied and the UV*-vis* spectra measured at all-time points are presented in SI (Additional file 1: Figure S10a, b). Moreover, data are consistent with what we reported in a previous study where silicon dioxide NPs have shown a similar behavior [24].

**Fig. 4:**
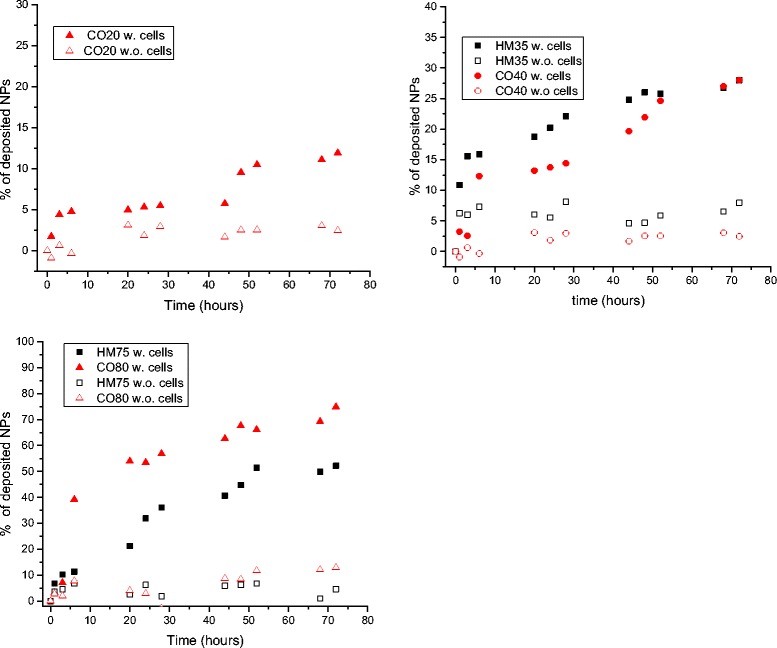
Percentage of NPs up-taken by or deposited on the cell monolayer (*filled markers*) and deposited on well plate surface (*empty markers*)

To compare the transport kinetics of the HM and CO sets of NPs, we calculated the area under the curve of each spectrum for all time points. The values were then normalized and subtracted from the values measured at t = 0 s in order to obtain the fraction of NPs taken up by the cells or bound to the cell membrane as a function of time. The results are presented in Fig. 4.

For all NP sizes, the curves show that the percentage of NPs deposited in presence of the cells is higher than the NPs percentage deposited on the well bottom without cells. The percentages of deposited NPs are size and time dependent. After 72 h, 28 and 52 % of the initial NPs concentration of HM35 and HM75 NPs respectively are reaching the cell monolayer, while the percentages are 12, 28 and 75 % for CO20, CO40 and CO80 NPs respectively. In cell-free conditions, percentages lower than 10 % are found for all NP types and sizes.

## Discussion

In this work, we measured the cellular dose i.e. the sum of the NPs taken up by the cells/or bound to the cell membranes. This dose is thus a function of the transport of the NPs towards the cell monolayers, the NP affinities with the cells and the capacity of the cells to take up the NPs [21].

The transport of NPs towards the cell monolayer is mainly driven by diffusion and gravitational sedimentation. The diffusion rate D is a function of the surrounding liquid properties and of the NP diameter:

4 $$ D = \frac{k_B.T\ }{3\ \pi \eta\ {D}_{NP}} $$

where k_B_ is the Boltzmann constant, T is media temperature, η is the cell culture medium dynamic viscosity and D_NP_ is the NP diameter.

The gravitational velocity can be written as:

5 $$ V = \frac{g\left({\rho}_{NP}-{\rho}_m\right){D}_{NP}^2\ }{18\eta } $$

where V is the settling velocity, g is the acceleration due to gravity, ρ_NP_ and ρ_m_ are respectively the mass density of the NPs and of the medium.

From Eqs. (4) and (5) it is possible to deduced the clear influence of the NP diameter and mass density on the transport. The diffusion rate is inversely proportional to the NP diameter and it is independent of the mass density. In contrast the velocity of sedimentation is proportional to the square of the NP diameter and to the difference of mass density between the NPs and the surrounding media. Therefore, the determination of the NP size, mass density and state of agglomeration when in contact with the serum proteins of the culture medium is essential in order to characterize their effective mode of transport.

Results in Table 1 show that considered two by two (HM15/CO20, HM30/CO40, HM75/CO80), the NPs have similar core sizes as measured by TEM but very different hydrodynamic diameters in water and cell culture conditions. The results of size characterization by DLS in water show that the hydrodynamic diameter is - as expected - higher than the core diameter measured by TEM and it is highly dependent on the stabilizing agent of the NPs. The hydration layer, as calculated in our approach, is the difference between the DLS and the TEM diameters. The diameter increase of around 5 nm for HM set of NPs results from the presence of a hydration layer that is reported to be few nm only for citrate-stabilized particles [32]. The increase of hydrodynamic diameter of around 10 nm observed for the commercial particles results from the thicker hydration layer formed around the NPs resulting from the presence of the surfactant stabilizer.

This effect is further augmented when NPs are in contact with serum proteins due to the formation of the protein corona. The increase of radii of NPs that range from 10 nm to 20 nm depending of the type of NPs, confirms the presence of a rather thick protein corona around the NPs (Table 2). This increase of diameter has a direct impact on the sedimentation time measured by CLS and the calculated apparent mass density of the formed complexes.

The CLS sedimentation time *t* can be expressed from Eq. (6) as:

6 $$ t=\frac{K}{\varDelta \rho {D}^{2\ }} $$

where K is the constant dependent on the viscosity of the gradient, the rotation speed and the radii of the rotating disks, ∆ρ is the difference in density between the NP complexes and the fluid and D is the diameter of the NP complex. The presence of the protein corona increases the diameter of the NP complex and, because the proteins have a lower density than the gold NPs, this results in a decrease of the overall apparent density of the complex. The increase of the sedimentation time of the NP complex versus bare NPs shows that the decrease of the mass density has more influence than the increase of diameter. This effect is relevant for the small and dense particles.

The apparent density of the NP-protein complexes has been calculated by using the NP diameters measured by DLS (Table 2). As expected, the results show an important decrease of the apparent density due to the presence of the protein corona (decrease of about 30 %) as compared to those obtained in water and obviously as compared to gold bulk density.

Transport time calculations for bare gold NPs show that the transport is diffusion driven for particles smaller than 40 nm and sedimentation driven for particles larger than 40 nm [12, 13]. To compare theoretically the time of transport between different NP complexes, the time needed by each type of NPs to travel 1 mm has been calculated using the diameters measured by DLS and the mass densities determined by CLS, therefore taking into account the presence of the protein corona. The results are grouped in Table 4.

**Table 4:** Time of transport for 1 mm calculated from DLS and CLS diameters and mass density of NMs in complete medium

Sample	D_DLS_ (nm)	Apparent density (g/cm^3^)	Sedimentation time (s) measured by CLS	Time of transport by diffusion (hours)	Time of transport by sedimentation (hours)
HM 35	69.7	3.3	42.1	17.3	37.0
HM75	101.9	6.2	10.3	25.3	7.6
CO20	35.8	4.3	138.0	8.9	97.2
CO40	66.5	5.0	31.5	16.5	23.3
CO80	116.7	6.7	7.1	28.9	5.3

The type of transport that drives the NPs toward the cell monolayer and the bottom of the wells can be deduced qualitatively by comparing the corresponding transport time. Table 4 results show that CO20 NPs (D_DLS_ = 35.7 nm) transport is mainly driven by diffusion (shorter time of transport by diffusion) whereas the larger particle transports (CO80 and HM75) are sedimentation driven (shorter time of transport by sedimentation). For the medium-size particles (HM35 and CO40) both diffusion and sedimentation contribute to the NPs transport; however sedimentation seems to be involved to a lower extent. One can note that the time of transport of both diffusion and sedimentation are in the same range. The main difference is that transport by sedimentation is mono directional toward the well bottom whereas diffusion is multi directional or directed toward the negative concentration gradient, and so can play a different role in the transport equilibrium. Since these times of transport are in the range of the duration of our experiment (72 h) and because the transport depends on the equilibrium between diffusion and sedimentation, these results show that most probably not all the particles will settle at the bottom of the wells particularly in the case of low binding affinity. Furthermore, the sedimentation times measured by CLS show that long sedimentation time corresponds to diffusion driven and short sedimentation time to gravitation driven transport.

As expected, the results of NP characterisation show that protein corona plays an important role in the NP transport as it modifies the physical properties of the NPs. To confirm this, qualitative protein corona determination has been performed by SDS PAGE. When NPs were incubated with fresh CCM, SDS PAGE analysis showed that the protein corona composition highly depends on the stabilizing agent (Additional file 1: Figure S6). Indeed, we observed a significant change in corona composition between the two series of NPs in particular through the appearance of band at 91 kDa for CO NP series. This is due most likely to the different NP surface properties i.e. different Zeta potential and different stabilizing agents.

In cell culture conditions, biomolecules secreted by the cells alter the biological identity of the protein corona influencing the NP agglomeration state and cell membrane affinity [8]. In this work, the protein corona identity is probably changing with time. Initially, in the very first hours, it is formed mainly by serum proteins contained in the cell culture medium and with time the identity changes due to possible interaction with the proteins and biomolecules secreted by the cells. SDS PAGE analysis performed on nanoparticles exposed to cell conditioned media showed some changes of the protein corona composition depending on the NP types (Additional file 1: Figure S7) but no major differences were observed as compared to gel analysis performed on NPs incubated with fresh medium. Moreover, the UV-vis spectra of the supernatant taken from the well containing NPs and cells measured for all time points show no significant changes in the spectrum profile shape suggesting that no NP agglomeration is occurring during the long term NP exposure. This suggests that in our experimental conditions, the transport of the NPs toward the bottom of the well with or without the presence of the cells should be similar and that the main difference in the fraction of deposited NPs is due to the higher binding affinity to the cell monolayer as compared to cell free wells.

The second important parameter that may influence the NPs transport and therefore NPs deposition is the NP-protein complex affinity with the cell membrane and the surface at the bottom of the well [21, 22]. This affinity has a crucial role since it has an influence on the direction of the gradient driven diffusion transport [18].

In this work, the fractions of the NPs deposited on and/or taken up by the cells are calculated by subtracting the concentration of NPs remaining in the supernatant at different time points from the initial concentration divided by this initial concentration. Using this approach it is not possible to distinguish between NPs taken up by the cells from those merely deposited on cell membrane.

The results show that the percentages of deposited NPs are higher in presence of the cells as well as being both size and time dependent. In cell-free conditions, the fractions deposited are also size and time dependent but are generally much smaller.

In the experiments without cells, the proteins present in the CCM cover not only the NPs to form the protein corona but also coat the bottom of the well forming a protein layer. Therefore, the main interactions, which occur, are between proteins deposited on the NPs and proteins deposited on the well bottom, leading to weak non-specific adsorption i.e. very low affinity. Assuming that the binding affinity resulting from protein-protein interactions is similar for all NPs sizes, the results show that the NPs transport has a large influence on the amount of deposited particles. For NPs whose transport is mainly diffusion driven (C020, HM35, CO40 corresponding to short time of transport by diffusion in Table 4), the NPs reaching the surface at the bottom of the well bind weakly to the surface, potentially creating an accumulation of NPs close to the well bottom. This accumulation results in a diffusion gradient toward the top of the well that limits the transport toward the bottom of the well. For larger NPs (CO80, HM75 corresponding to short time of transport by sedimentation in Table 4), this transport limitation is attenuated since the transport by sedimentation becomes dominant resulting in a slight increase of the fraction of deposited NPs. The poor affinity and the limited transport rate, nevertheless, result in a small amount of particles becoming bound to the bottom of the well (from 2 to 10 % for respectively CO20 and CO80).

For all particles sizes and types, the kinetic of deposition or cellular uptake is found to be faster during the first hours and tends to slow down after 10 h. Assuming that the initial concentration of the NPs is uniform along the height of the liquid column, the transport of NPs can be divided in two phases. For short exposure duration (phase 1 i.e. uptake phase), the NPs in close proximity of the cell monolayer are rapidly internalized by the cells creating a negative gradient of concentration toward the cell monolayer. This gradient of concentration tends to favour the diffusion the NPs towards the bottom of the well. The diffusion is dominant for the smaller NPs (CO20, HM35 and CO40). For larger particles the transport by diffusion sums with the transport by sedimentation increasing the fraction of nanoparticles reaching the cell monolayer. However, the uptake may become transport limited, in particular, for NPs having sizes that do not favour neither sedimentation nor transport by diffusion.

In the second phase (phase 2: deposition phase) when the NPs uptake tends to reach saturation, the preferential mode of interaction is the non-specific i.e. low affinity NP deposition on the cell monolayer. The rate of deposition tends to be constant and depends on the NP transport and the binding affinity. The decrease of the uptake results in an accumulation of NPs in close proximity to the cell monolayer creating a negative concentration gradient toward the top of the well forcing the net diffusion in this direction and limiting the deposition of the NPs for small particles [18]. For larger particles, the main transport is sedimentation driven so is not a limiting factor for the NP deposition. Of course this equilibrium depends on the density and size of the particles.

Concerning the effect of the stabilisation agent, the NPs deposition is found to be higher for CO80 than for HM75 even if they have similar size, density and therefore transport properties. This difference is most likely due to the different nature of the protein corona and consequently different affinity with the cell membrane. Nevertheless, such a difference is not observed for CO40 and HM 35 NPs. Although a clear difference in the protein corona composition is observed, as showed in Additional file 1: Figures S6-S7, we could not find a clear trend of the influence of the stabilizing agent on the cellular dose.

The amount of NPs deposited at each time-point has been calculated from the initial number of NPs incubated with the cells. The results are presented in Table 5.

**Table 5:** Calculated NPs number deposited on a cell

	CO20	CO40	CO80	HM35	HM75
Initial Nb. of NPs/ml	7.20 10^11^	7.20 10^10^	7.80 10^9^	7.30 10^10^	5.70 10^9^
NPs/well	5.76 10^10^	5.76 10^9^	6.24 10^8^	5.84 10^9^	4.56 10^8^
NPs/cell 6 h	3.7 10^4^	1.0 10^4^	1.1 10^3^	1.3 10^4^	7.2 10^2^
NPs/cell 72 h	8.2 10^4^	1.7 10^4^	6.7 10^3^	1.67 10^4^	3.4 10^3^

**Fig. 5:**
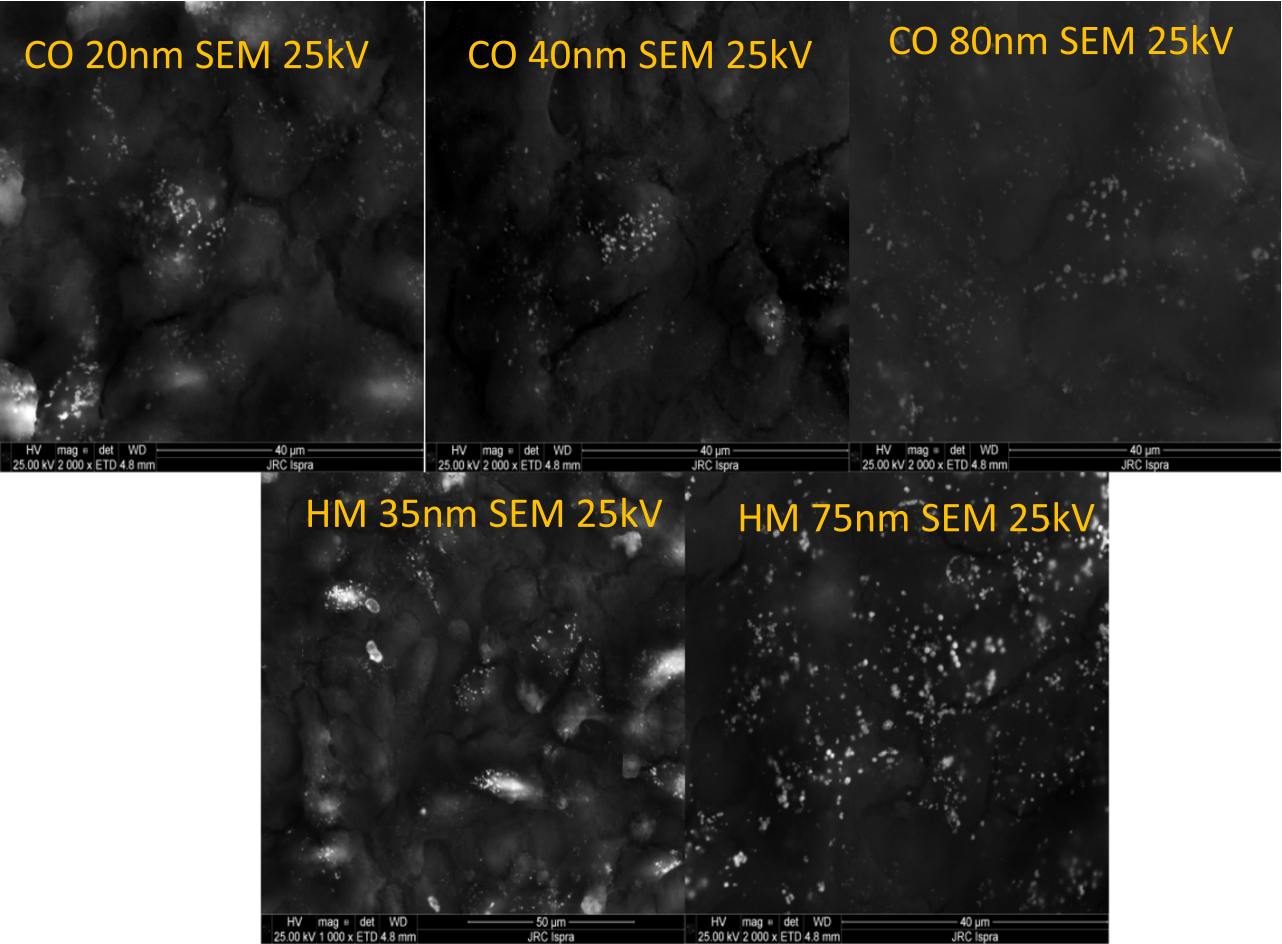
SEM images of cell monolayer incubated for 72 h with the 2 sets of NPs

Studies of gold NPs uptake found in literature are often based on experiments having exposure duration between 6 and 12 h [22, 33]. In these works, uptake saturation is observed after few hours and the number of particles taken up by the cells is in the range of a few thousand per cell for concentrations of 16 μM [22]. These studies also usually include several washing steps in order to remove as much as possible of the NPs bound on the cell membrane. In our studies, because the objective was to determine the cellular dose, the wells were not washed before performing UV-*vis* measurements. After six hours of exposure the numbers of particles taken up and deposited onto the cells are in reasonable agreement with those obtained in the literature taking in account different experimental conditions (different NPs concentration and liquid height). In order to illustrate the deposition of the NPs onto the cell monolayers, SEM images of cells after 72 h of exposure are presented in Fig. 5. These images show clearly that a large number of NPs is deposited on the cells membranes forming clusters of about 500 nm for larger NPs. The identity of the NPs is confirmed by the elemental analysis performed by EDX (Additional file 1: Figure S12).

**Fig. 6:**
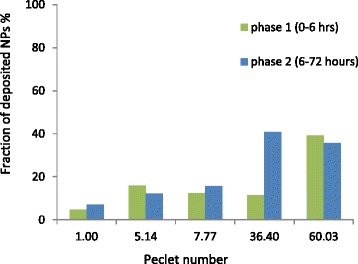
Fraction of deposited NPs as a function of the Peclet number

To confirm the influence of transport on the fraction of deposited NPs, we calculated the Peclet number (Pe) which represents the ratio between the sedimentation driven to diffusion driven transport [34] (Table 6):

**Table 6:** Peclet number calculated from experimental data

	HM35	HM75	CO20	CO40	CO80
Peclet number	5.14	36.40	1.00	7.77	60.03

7 $$ Pe = \frac{V.h}{D} $$

Where V and D are the velocity of sedimentation and the diffusion calculated using the experimental measurement results and h is the liquid height (5.5 mm in our experiments).

The fraction of deposited NPs has been plotted as a function of the Peclet number for time points of 6 and 72 h (Fig. 6).

For the 6 h time point (phase 1), no clear relation between the transport conditions (Peclet number) and the fraction of deposited NPs can be deduced from this curve. This means that for short time of exposure, the fraction of deposited or up taken NPs is mainly driven by the affinity with the cell membrane and not by the transport. However, for longer time of exposure (phase 2), the fraction of deposited NPs increases steadily with the Peclet number showing that gravitational driven transport has the greatest influence on the NPs deposition for larger size NPs. Nevertheless, results obtained with the CO80 suggest that for higher Peclet number (larger NPs), Phase 1 and 2 are somehow merged due to the high rate of sedimentation. Whereas the NPs uptake tends to saturate and is limited by the cell uptake capacity, the amount of NPs deposited on the cellular membrane increases with the initial concentration and depends very much of their transport mode [35, 36].

The present method that integrates the extended NP characterisation and cellular dose determination by UV-vis measurement could be applied to any type of nanoparticles having a rather intense surface plasmon resonance and negligible solubility. However, it should be noted that the determination of the cellular dose of soluble nanoparticles, in particular metal oxide and Ag NPs is very challenging. Indeed, NP dissolution results in a decrease of the NP concentration and diameter and in an increase of the dissolved materials in the medium as a function of the time. Depending of the culture media, dissolution may also result in some instability leading to NPs agglomeration [37]. Therefore, it is clear that the dissolution may have a strong influence the mode of transport of the NPs. In the case of soluble NPs, the characterisation techniques used in this work to determine the NP physical properties must be thus complemented by ICP-MS measurements to determine the amount of dissolved material and the dissolution rate. The knowledge of these parameters will make it possible to determine the cellular dose that affects the cell behaviour including for instance the dose of Ag ion released, which is known to be toxic [38, 39]. The method is as best suited to very stable nanoparticles since it is based on the monitoring of the UV-vis spectra measured from the supernatant. Agglomeration would complicate drastically the analysis of the data by changing the NP optical properties. In the case of agglomerates, the volumetric centrifugation method combined with ICP-MS presented in reference [17] is more suitable. Systematic studies, as presented in this work, should contribute to the validation of numerical models. Indeed, the knowledge of important parameters such as density and size of NPs complexes combined with experimental data of deposition kinetics performed with and without cells can be advantageously used in published numerical models [15, 16, 18]. In these models, one of the most important parameters which must be known is the cell membrane affinity which is necessary to defining the boundary condition that simulates the cell monolayer. Determining this parameter is very challenging but appropriate application of the techniques described in this work could provide the means to evaluate cell membrane affinity coefficients by fitting the experimental curves of deposition kinetics [13].

## Conclusion

In summary, this study show that the deposition rate of gold NPs on the cell monolayer strongly depends on their physical properties (size and mass density) and transport mode; the smaller the particles are, the lower is the fraction of deposited NPs. The presence of the cells is essential to study the NP transport in vitro, since the NP affinity to the cells plays a important role on the overall transport to the bottom of the wells. Because the kinetics of NPs is composed of 2 phases, the experimental factors such as properties of the NP-protein complex, liquid heights in the wells and duration of the exposure have a clear effect on cellular dose. The choice of the initial NP concentration used in the in vitro assay is of importance since excessively high concentrations may lead to an overload of NPs on the cell membranes thus modify the nature of interactions of NPs with the cells [10, 18, 35, 36]. Such systematic studies presented in this work will help to develop robust, standardized and reliable in vitro assays for NM toxicity screening*.*

## Methods

### Synthesis and characterization of Au NPs

Commercial gold NPs were purchased from Sigma-Aldrich (3050 Spruce Street, Saint Louis, MO 63103, USA) and were produced by the CytoDiagnostics, Inc. All three sizes of Au NPs (20 nm Cat. Nr.741695, 40 nm Cat. Nr.741981 and 80 nm Cat. Nr.742023) were stabilized suspensions in citrate buffer. However the composition of the stabilizer was not disclosed. The NPs concentrations supplied were 7.210^+11^ NPs/ml for CO 20 nm, 7.210^+10^ NPs/ml for CO 40 nm and 7.8.10^+9^ NPs/ml for CO 80 nm.

Synthesis of HM15 NPs was carried out by modification of the procedure described by Turkevich et al., [40] in which the gold NPs are produced by the reduction of the gold salt by the sodium citrate that acts as a reducing agent and stabilizer. In this work, the solution was heated up using a specialized microwave apparatus (Discover S by CEM corporation) to ensure a highly reproducible rapid heating. In this method, 5 ml of tetrachloroauric acid trihydrate 0.01 M (HAuCl_4_ · 3H_2_O) (Sigma-Aldrich) was dissolved in 95 ml of water. The solution was rapidly heated up and hold at 97 °C for 5 min using a maximum microwave power of 250 W under vigorous mechanical stirring. In these conditions, 2.5 ml of trisodium citrate dihydrate 0.1 M (Sigma-Aldrich) was added to the solution and kept at 100 °C for further 20 min. Afterwards, the solution was rapidly cooled down to 40 °C.

The synthesis of HM 35 NPs was carried out by a regrowth method of 15 nm Au NPs. 95 ml of MilliQ water were left to stir at 60 °C until equilibrium was reached. Then, 2.8 ml of sodium citrate dihydrate (0.1 M) and 0.42 ml of 200 mM of sodium hydroxide (Sigma-Aldrich) were added to the water solution. After 30 min, 2.24 ml of HAuCl_4_ · 3H_2_O 10 mM and 2.6 ml of 12 nm gold NPs were added to the solution, under vigorous stirring. The solution was left to react for 48 h at 60 °C. The nominal concentration of gold in NPs dispersion was 0.24 mM. The synthesis of HM 75 NPs was carried out by a regrowth method of 40 nm NPs. 70 ml of MilliQ water were left to stir at 60 °C and then 2.8 ml of sodium citrate dihydrate (0.1 M) and 0.42 ml of 200 mM of sodium hydroxide (Sigma-Aldrich) were added to the water solution. After 30 min, 1.25 ml of HAuCl_4_ · 3H_2_O 10 mM and 25 ml of 40 nm Au NPs were added to the solution, under vigorous stirring. The reaction time was 48 h at 60 °C.

### Nanoparticles characterization

#### Dynamic Light Scattering (DLS) measurements

The DLS measurements were performed with Zetasizer Nano ZS system Malvern Instruments (Malvern, UK) using disposable micro cuvettes (500 μl of NPs,) with the material refractive index of 0.20 and material absorption of 3.320. Gold NPs were suspended in complete cell culture medium at 37 °C at concentrations of 60 and 40 μM for respectively CO and HM sets of NPs. The following measurement settings were used: dispersant refractive index of 1.330, viscosity (cP) of 0.8872, temperature of measurement 25 °C, duration of measurement of 60s and measurement position of 3 mm. Data are reported as Z-average or as intensity based particle size distribution by using respectively cumulant analysis and multimodal analysis (multi exponential- Malvern, DTS algorithm). All DLS experiments were performed in triplicates.

### Transmission electronic microscopy analysis

TEM samples were prepared by depositing 5 μL of the solution containing the NPs on carbon-coated TEM grids (carbon type-B, 200 mesh copper grids, supplied by Ted Pella, Inc.) and dried in air. High-resolution TEM imaging was performed on a JEOL 2100 microscope operated at an acceleration voltage of 200 kV. Digital images were analysed with the ImageJ software (available at http://rsb.info.nih.gov/ij/). The NP size distribution was determined by image processing of several TEM images in order to evaluate at least 100 isolated primary particles using the interface Particle Size Analyzer (PSA) macro for ImageJ.

### Centrifuge liquid sedimentation (CLS) analysis

Centrifugal Liquid Sedimentation measurements (DC24000UHR model by CPS Instruments Measurements) were performed at disc speed was 22,000 rpm. Sucrose density gradient range was 8 wt % - 24 wt % and injected sample volume was 100 μL. Each sample injection was preceded by a calibration step performed using certified poly (vinyl chloride) particle size standards with mean size of 380 nm. CLS experiments were performed in triplicate. The dispersion of the NPs in cell culture media was done after preheating the media at 37 °C degrees in the water bath without any sonication (NP concentrations : 60 and 40 μM for respectively CO and HM sets of NPs).

### Contact angle measurements

The contact angle with water was measured using a Digidrop Contact Angle meter (GBX, France). The two stabilizer (surfactant stabilizer for commercial NPs and citrate for in house synthetized NPs) were spotted on a gold surface to form of confluent film. Then, a 0.5 μL water droplet was deposited on the surface of the surface and the contact angle measured.

### SDS page analysis

The NPs were incubated with cell culture medium containing serum proteins for few minutes (t = 0) and 72 h (t = 72). The NPs mix was centrifuged 30 min at 12000RPM and the supernatant was carefully removed. The NPs pellet was subsequently washed three times with 1× PBS (Gibco) and centrifuged at 12000RPM. The final pellet was suspended in 20uL 2× Laemmli Buffer (Sigma) and incubated at 95 deg for five minutes. After a short spin down the supernatant was loaded in 12 % SDS Polyacrylamide gel and was run at 110 V, 25 mA in 1× SDS Running Buffer. After electrophoresis, the gel was Coomassie stained.

### Cell monolayer integrity assessment

Adenocarcinoma human alveolar basal epithelial cells (A549) were maintained in 75 cm^2^ flasks (Corning) using HAM-F12 + Glutamax™-I medium (Life Technologies) supplemented with 10 % heat-inactivated foetal bovine serum (Life Technologies), 10,000 U Penicillin/Streptomycin (Life Technologies) and 0.5 % (v/v) Hepes (Life Technologies) under normal cell culture conditions (37 °C; 5 % CO_2_; 95 % humidity). For the assessment of cell monolayer integrity after Au NPs exposure, the cells were seeded in 96 well plates (Corning™ Costar™ 96-Well Black) and cultured until confluence was reached. Cell monolayers were exposed to different sizes and types of Au NPs at a final concentration of 60 and 40 μM of respectively CO and HM sets of NPs in CCM for 72 h (11 time points were assessed). Afterwards, cells were stained with Hoechst 8 μM (Molecular Probes) and Propidium Iodide 3 μM (Sigma) for 15 min at 37 °C in order to assess the cell viability. Cells were analyzed by High Content Analysis using IN Cell Analyzer 2200 (GE Healthcare). During acquisition, a minimum of 9 fields per well were imaged using a 10× objective. Data analysis was performed on the IN Cell Investigator Software (GE Healthcare) using in house developed protocols with a minimum of 25,000 cells analyzed for each condition. Cell viability was calculated by determining the number of live cells, i.e. total (Hoechst 33342 positive) cells minus dead (propidium iodide positive) cells, normalized by the number of viable cells in control wells.

### Measurement of gold NP deposition

A549 cells were plated at a density of 15000 cells per well on 96 well plates and cultured for 72 h until a uniform monolayer of cells was formed. Gold NPs were suspended in complete cell culture medium at concentrations of 60 and 40 μM for respectively CO and HM sets of NPs. 200 μl of the NP suspension was added to the wells (liquid height = 5.5 mm) of a 96-well plate (empty or containing a monolayer of A549 cells, ~70000 cells/well). Phenol red free culture medium was used in order to avoid colorimetric interferences with the NP plasmonic peaks particularly for HM75 and CO80 NPs. All gold NP exposures and measurements were performed in triplicate for each time point. UV-*vis* spectroscopy was used to study the kinetics of NP deposition in culture medium. At given time points (from t = 0 up to t = 72 h) the medium was gently transferred to another 96 well plate and the UV-*vis* absorbance spectra of the supernatant wells were measured in a multi-well spectrophotometer (FLUOstar Omega, BMG labtech, Germany).

Three separate spectra corresponding to each individual time point were acquired and then the measurements were averaged. Baseline subtraction was performed for each triplicate average and the surface area of the spectrum was calculated. The baseline was the UV-*vis* spectrum measured with cell culture medium from unexposed cells.

After transferring the medium containing NPs, cells were washed in 200 μl of PBS. The UV-*vis* absorbance measurements of the washing medium results in a weak signal close to the detection limit of the spectrometer.

The number of cells has been determined first by the automatic counting of the Hoechst 33342 stained nuclei from a square area of the bottom of each well of the 96 well plates using the IN Cell Analyser 2200 Imaging System. Additional file 1: Figure S11 shows that the number of cells was constant in all the experiments and did not differ from the untreated control. The cell nuclei were then counted manually in a number of representative wells over the whole area of the well including side wedges which were not accessible for the IN Cell automatic system. Nuclei were counted in 3 representative wells. The average value of cells of 70,000 cells/well was then used for calculating the NP number by cells.

### Scanning electron microscopy

To perform SEM imaging, the cells were cultured on a silicon wafer coated with a Poly Acrylic acid layer. A 150-nm thick pAA layer was deposited on silicon wafer by plasma processing [41]. The coated wafer was then cut in 10 ×10 mm chips, rinsed in ultrapure water and sterilized under UV light for 1 h. After sterilization the chips were immersed in 1× PBS for 1 day in order to check the film stability. The A549 cells were seeded at a concentration of 50 000 cell/ml on 24 well plates containing chips on the bottom. After 72 h the silicon chips containing A549 cells samples were transferred to new 24 well plates and were exposed to 60 and 40 μM of respectively CO and HM sets of NPs in CCM for 72 h. Then the cells were washed with 1× PBS, fixed in 4 % formaldehyde, washed three times with PBS, then with distilled water, and dehydrated in increasing concentrations (25, 50, 75 and 100 %) of ethanol.

SEM measurements on gold NPs exposed cells were performed by a FEI NOVA 600, Dual Beam, using an acceleration voltage ranging from 5 KeV and 25 KeV and acquiring secondary electrons. The same scanning area was imaged at increasing electron acceleration voltage, until reaching a good contrasted image between the gold NPs and the cells. Energy-Dispersive X-ray Spectroscopy (EDX) has been carried out in-situ using an EDAX analyser (AMETEK BV, The Netherlands) with element spectral resolution and sensitivity down to the carbon element. Elemental analysis maps were acquired by acquiring first a secondary electron map at 20 KeV and the X-ray elemental spectrum of the scanned region. Different elements were detected, including gold (main peak at 2.12 KeV, corresponding to the Ma line). Then the elemental map was generated fixing the energy at the Au peak and scanning the intensity of the generated X-rays over the field-of-view.

## Additional file

Additional file 1: Figure S1. a, b: HR-TEM images and quantitative particle size distribution analysis **Figure S2** a, b: CLS size distribution intensity curves. **Figure S3** a, b: DLS size distribution intensity curves for the two sets of particles in water and CCM. **Figure S4** a, b: CLS sedimentation times for the two sets of NPs in water and CCM. **Figure S5:** UV*-vis* measurement for the two sets of NPs in water and CCM. **Figure S6:** SDS page images of protein corona isolated from CO and HM series NPs at t = 0 and t = 72 h. **Figure S7:** SDS page images of protein corona isolated from CO and HM series NPs at t = 0 and t = 72 h. Cell culture medium was conditioned with A549 for 72 h. **Figure S8:** Cell monolayer integrity monitoring by statistical analysis of viability measurements of the cell monolayer exposed to different gold NPs. **Figure S9** a, b, c: Raw UV-*vis* spectra measured in CCM at different time points. **Figure S10**, b: UV-*vis* spectra measured for the two sets of NPs after baseline subtraction and normalization. **Figure S11:** Number of cells per well plotted versus time point counted in automatic with the IN Cell Analyser 2200 Imaging System of the Hoechst stained nuclei from a square area of the bottom of the 96 well. **Figure S12:** SEM picture on the A549 cells treated with different NPs sizes on the left. On the rigth are gold mapping performed with EDX for NPs from the selected regions of the SEM pictures. This material is available free of charge *via* the Internet at http://particleandfibretoxicology.com. (PPTX 8652 kb)

## Abbreviation

NPs: Nanoparticles
NMs: Nanomaterials
DLS: Dynamic light scattering
CLS: Centrifugal liquid sedimentation
CCM: Complete culture medium
TEM: Transmission electronic microscopy
SPR: Surface plasmon resonance
SDS-PAGE: Sodium Dodecyl Sulphate Polyacrylamide gel electrophoresis
Pe: Peclet number
EDX: Energy-dispersive X-ray spectroscopy

## References

[1] Vance ME, Kuiken T, Vejerano EP, McGinnis SP, Hochella MF, Rejeski D, Hull MS (2015). Nanotechnology in the real world: redeveloping the nanomaterial consumer products inventory. Beilstein J Nanotechnol.

[2] Krug HF, Wick P (2011). Nanotoxicology: an interdisciplinary challenge. Angew Chem Int Ed Engl.

[3] Krug HF (2014). Nanosafety research—are we on the right track?. Angew Chem Int Ed.

[4] Bouwmeester H, Lynch I, Marvin HJ, Dawson KA, Berges M, Braguer D, Byrne HJ, Casey A, Chambers G, Clift MJ, Elia G, Fernandes TF, Fjellsbø LB, Hatto P, Juillerat L, Klein C, Kreyling WG, Nickel C, Riediker M, Stone V (2011). Minimal analytical characterization of engineered nanomaterials needed for hazard assessment in biological matrices. Nanotoxicology.

[5] Editorial (2012). Join the discussion. Nat Nanotechnol.

[6] Monopoli MP, Åberg C, Salvati A, Dawson KA (2012). Biomolecular coronas provide the biological identity of nanosized materials. Nat Nano.

[7] Lynch I, Salvati A, Dawson KA (2009). Protein-nanoparticle interactions: What does the cell see?. Nat Nano.

[8] Albanese A, Walkey CD, Olsen JB, Guo H, Emili A, Chan WCW (2014). Secreted biomolecules alter the biological identity and cellular interactions of nanoparticles. ACS Nano.

[9] Walkey CD, Chan WC (2012). Understanding and controlling the interaction of nanomaterials with proteins in a physiological environment. Chem Soc Rev.

[10] Lison D, Vietti G, van den Brule S (2014). Paracelsus in nanotoxicology. Part Fibre Toxicol.

[11] Oberdörster G (2012). Nanotoxicology: in vitro-in vivo dosimetry. Environ Health Perspect.

[12] Teeguarden JG, Hinderliter PM, Orr G, Thrall BD, Pounds JG (2007). Particokinetics in vitro: dosimetry considerations for in vitro nanoparticle toxicity assessments. Toxicol Sci.

[13] Cohen J, DeLoid G, Demokritou PA (2015). Critical review of in vitro dosimetry for engineered nanomaterials. Nanomedicine.

[14] Cho EC, Zhang Q, Xia Y (2011). The effect of sedimentation and diffusion on cellular uptake of gold nanoparticles. Nat Nano.

[15] Hinderliter PM, Minard KR, Orr G, Chrisler WB, Thrall BD, Pounds JG, Teeguarden JG (2010). ISDD: a computational model of particle sedimentation, diffusion and target cell dosimetry for in vitro toxicity studies. Part Fibre Toxicol.

[16] Cohen J, Teeguarden J, Demokritou P (2014). An integrated approach for the in vitro dosimetry of engineered nanomaterials. Part Fibre Toxicol.

[17] DeLoid G, Cohen JM, Darrah T, Derk R, Rojanasakul L, Pyrgiotakis G, Wohlleben W, Demokritou P (2014). Estimating the effective density of engineered nanomaterials for in vitro dosimetry. Nat Commun.

[18] DeLoid GM, Cohen JM, Pyrgiotakis G, Pirela SV, Pal A, Liu J, Srebric J, Demokritou P (2015). Advanced computational modeling for in vitro nanomaterial dosimetry. Part Fibre Toxicol.

[19] Liu R, Liu HH, Ji Z, Chang CH, Xia T, Nel AE, Cohen Y (2015). Evaluation of toxicity ranking for metal oxide nanoparticles via an in vitro dosimetry model. ACS Nano.

[20] Hirsch V, Kinnear C, Rodriguez-Lorenzo L, Monnier CA, Rothen-Rutishauser B, Balog S, Petri-Fink A (2014). In vitro dosimetry of agglomerates. Nanoscale.

[21] Wilhelm C, Gazeau F, Roger J, Pons JN, Bacri JCI (2002). Interaction of anionic superparamagneticnanoparticles with cells: kinetic analyses of membrane adsorption and subsequent internalization. Langmuir.

[22] Chithrani BD, Ghazani AA, Chan WC (2006). Determining the size and shape dependence of gold nanoparticle uptake into mammalian cells. Nano Lett.

[23] Dykman LA, Khlebtsov NG (2014). Uptake of engineered gold nanoparticles into mammalian cells. Chem Rev.

[24] Halamoda-Kenzaoui B, Ceridono M, Colpo P, Valsesia A, Urbán P, Ojea-Jiménez I, Gioria S, Gilliland D, Rossi F, Kinsner-Ovaskainen A (2015). Dispersion behaviour of silica nanoparticles in biological media and its influence on cellular uptake. PLoS One.

[25] Cho EC, Liu Y, Xia Y (2010). A simple spectroscopic method for differentiating cellular uptakes of gold nanospheres and nanorods from their mixtures. Angew Chem Int Ed Engl.

[26] Dykman LA, Khlebtsov NG (2012). Gold nanoparticles in biomedical applications: recent advances and perspectives. Chem Soc Rev.

[27] Alkilany AM, Murphy CJ (2010). Toxicity and cellular uptake of gold nanoparticles: what we have learned so far?. J Nanopart Res.

[28] Bell NC, Minelli C, Shard AG (2013). Quantitation of IgG protein adsorption to gold nanoparticles using particle size measurement. Anal Methods.

[29] Kamiti M, Boldridge D, Ndoping LM, Remsen EE (2012). Simultaneous absolute determination of particle size and effective density of submicron colloids by disc centrifuge photosedimentometry. Anal Chem.

[30] Krpetić Z, Davidson AM, Volk M, Lévy R, Brust M, Cooper DL (2013). High-resolution sizing of monolayer-protected gold clusters by differential centrifugal sedimentation. ACS Nano.

[31] Capomaccio R, Jimenez I, Colpo P, Gilliland D, Ceccone G, Rossi F, Calzolai L (2015). Determination of the structure and morphology of gold nanoparticle–HSA protein complexes. Nanoscale.

[32] Falabella JB, Cho TJ, Ripple DC, Hackley VA, Tarlov MJ (2010). Characterization of gold nanoparticles modified with single-stranded DNA using analytical ultracentrifugation and dynamic light scattering. Langmuir.

[33] Brandenberger C, Mühlfeld C, Ali Z, Lenz A, Schmid O, Parak WJ, Gehr P, Rutishauser B (2010). Quantitative evaluation of cellular uptake and trafficking of plain and polyethylene glycol coated gold nanoparticles. Small.

[34] Mahnama A, Ghorbaniasl G, Allaei S, Nourbakhsh A (2014). Semi-analytical solution for the in-vitro sedimentation, diffusion and dosimetry model: surveying the impact of the peclet number. Colloids Surf B Biointerfaces.

[35] Wittmaack K (2011). Excessive delivery of nanostructured matter to submersed cells caused by rapid gravitational settling. ACS Nano.

[36] Lison D, Thomassen LC, Rabolli V, Gonzalez L, Napierska D, Seo JW, Kirsch-Volders M, Hoet P, Kirschhock CE, Martens JA (2008). Nominal and effective dosimetry of silica nanoparticles in cytotoxicity assays. Toxicol Sci.

[37] Tejamaya M, Römer I, Merrifield R, Lead J (2012). Stability of citrate, PVP, and PEG coated silver nanoparticles in ecotoxicology media. Environ Sci Technol.

[38] Loza K, Diendorf J, Sengstock C, Ruiz-Gonzalez L, Gonzalez-Calbet J, Vallet-Regi M (2014). The dissolution and biological effects of silver nanoparticles in biological media. J Mater Chem B.

[39] Xia T, Kovochich M, Liong M, Mädler L, Gilbert B, Shi H (2008). Comparison of the mechanism of toxicity of zinc oxide and cerium oxide nanoparticles based on dissolution and oxidative stress properties. ACS Nano.

[40] Turkevich J, Stevenson PC, Hillier J. A study of the nucleation and growth processes in the synthesis of colloidal gold. Discuss Faraday Soc. 1951;55–75.

[41] Brétagnol F, Valsesia A, Ceccone A, Colpo P, Gilliland D, Ceriotti L, Hasiwa M, Rossi F (2006). Surface functionalization and patterning techniques to design interfaces for biomedical and biosensor applications. Plasma Process Polym.

