# Erratum to: Quantification of the cellular dose and characterization of nanoparticle transport during in vitro testing

**DOI:** 10.1186/s12989-016-0180-2

**Published:** 2016-12-09

**Authors:** Grigore Rischitor, Mariantonietta Parracino, Rita La Spina, Patricia Urbán, Isaac Ojea-Jiménez, Elena Bellido, Andrea Valsesia, Sabrina Gioria, Robin Capomaccio, Agnieszka Kinsner-Ovaskainen, Douglas Gilliland, François Rossi, Pascal Colpo

**Affiliations:** 1 European Commission Joint Research Centre, Institute for Health and Consumer and Protection, Nanobiosciences Unit, Via E. Fermi 2749, 21027 Ispra, VA Italy; 2 Present Address: Nanoimmunotech S.L., Av. de la Autonomía, 50003 Zaragoza, Spain

## Erratum

After the publication of this work [1] it was noticed that the author name ‘Patricia Urbán’ was incorrectly spelt as ‘Patrizia Urbán’. This has now been corrected in the author list.
