# Supplementary material for: Quantification of the cellular dose and characterization of nanoparticle transport during in vitro testing
Source: Part Fibre Toxicol. 2016 Aug 24;13:47. doi: 10.1186/s12989-016-0157-1 (PMC4995798; doi:10.1186/s12989-016-0157-1)
Supplement: Additional file 1: Figure S1. — a, b: HR-TEM images and quantitative particle size distribution analysis Figure S2 a, b: CLS size distribution intensity curves. Figure S3 a, b: DLS size distribution intensity curves for the two sets of particles in water and CCM. Figure S4 a, b: CLS sedimentation times for the two sets of NPs in water and CCM. Figure S5: UV-vis measurement for the two sets of NPs in water and CCM. Figure S6: SDS page images of protein corona isolated from CO and HM series NPs at t = 0 and t = 72 h. Figure S7: SDS page images of protein corona isolated from CO and HM series NPs at t = 0 and t = 72 h. Cell culture medium was conditioned with A549 for 72 h. Figure S8: Cell monolayer integrity monitoring by statistical analysis of viability measurements of the cell monolayer exposed to different gold NPs. Figure S9 a, b, c: Raw UV-vis spectra measured in CCM at different time points. Figure S10, b: UV-vis spectra measured for the two sets of NPs after baseline subtraction and normalization. Figure S11: Number of cells per well plotted versus time point counted in automatic with the IN Cell Analyser 2200 Imaging System of the Hoechst stained nuclei from a square area of the bottom of the 96 well. Figure S12: SEM picture on the A549 cells treated with different NPs sizes on the left. On the rigth are gold mapping performed with EDX for NPs from the selected regions of the SEM pictures. This material is available free of charge via the Internet at http://particleandfibretoxicology.com. (PPTX 8652 kb) [file 12989_2016_157_MOESM1_ESM.pptx]

## Slide 1
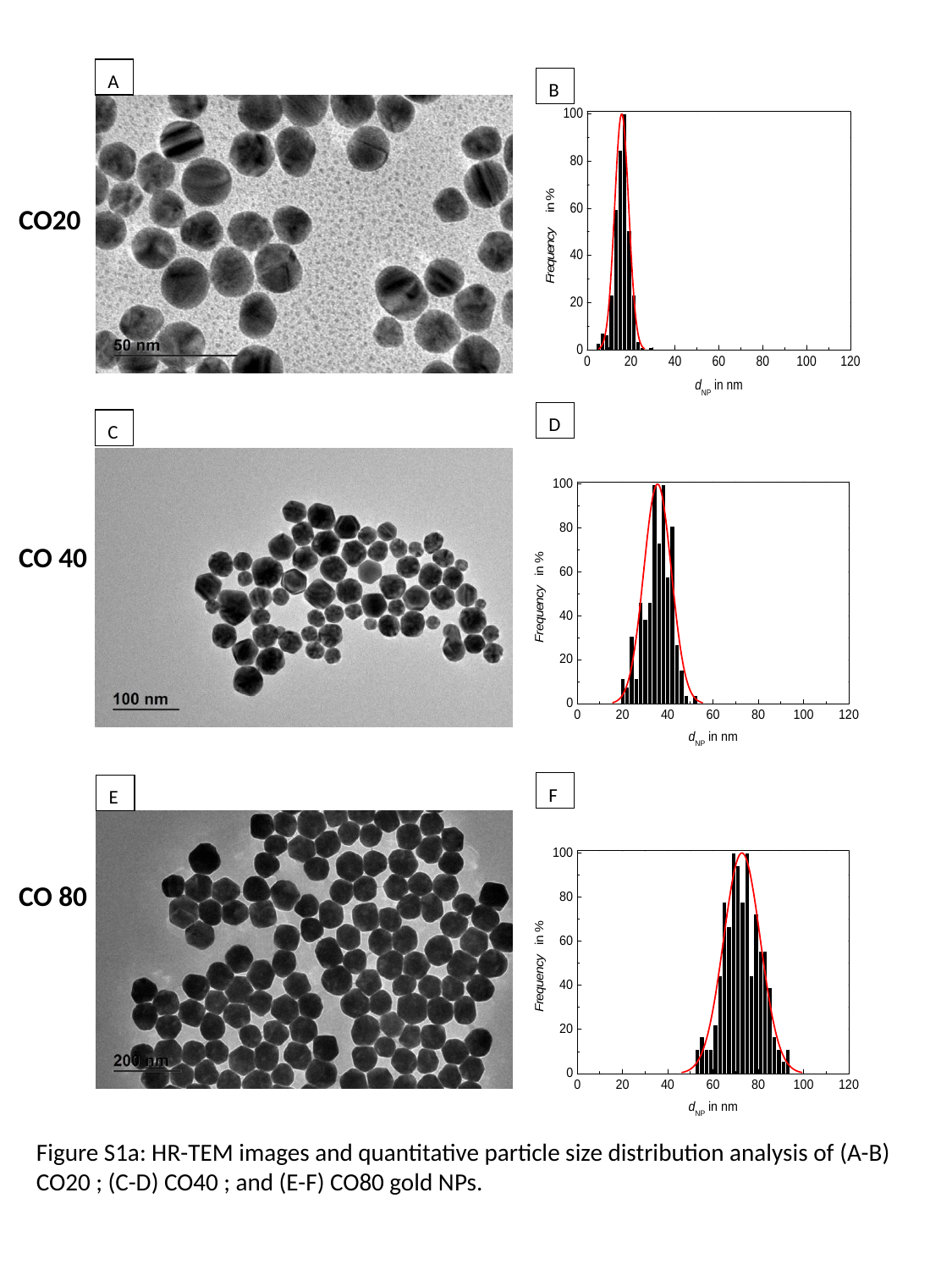

A
B
CO20
CO 40
CO 80
D
C
F
E
Figure S1a: HR-TEM images and quantitative particle size distribution analysis of (A-B) CO20 ; (C-D) CO40 ; and (E-F) CO80 gold NPs.

## Slide 2
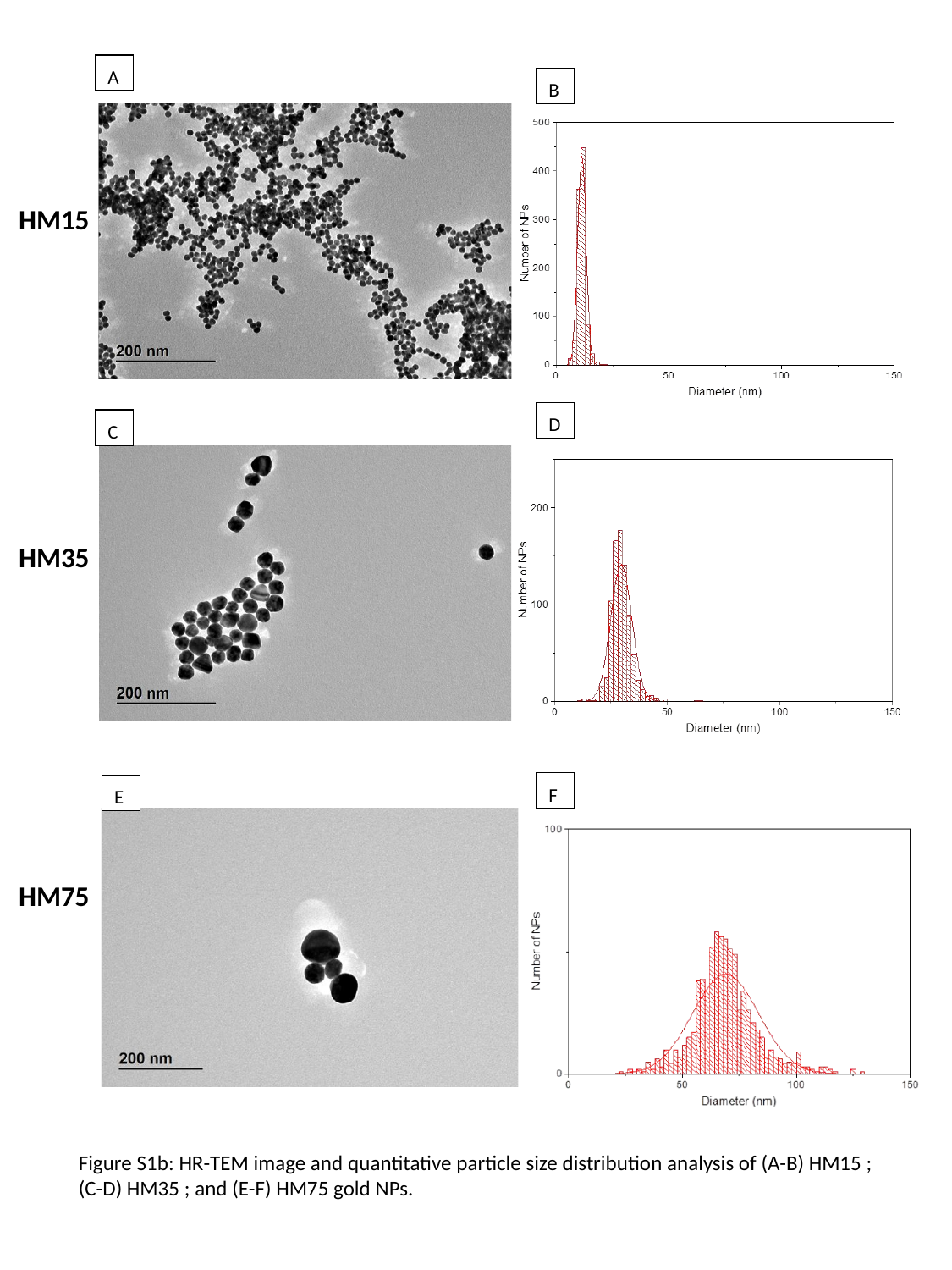

A
B
HM15
HM35
HM75
D
C
F
E
Figure S1b: HR-TEM image and quantitative particle size distribution analysis of (A-B) HM15 ; (C-D) HM35 ; and (E-F) HM75 gold NPs.

## Slide 3
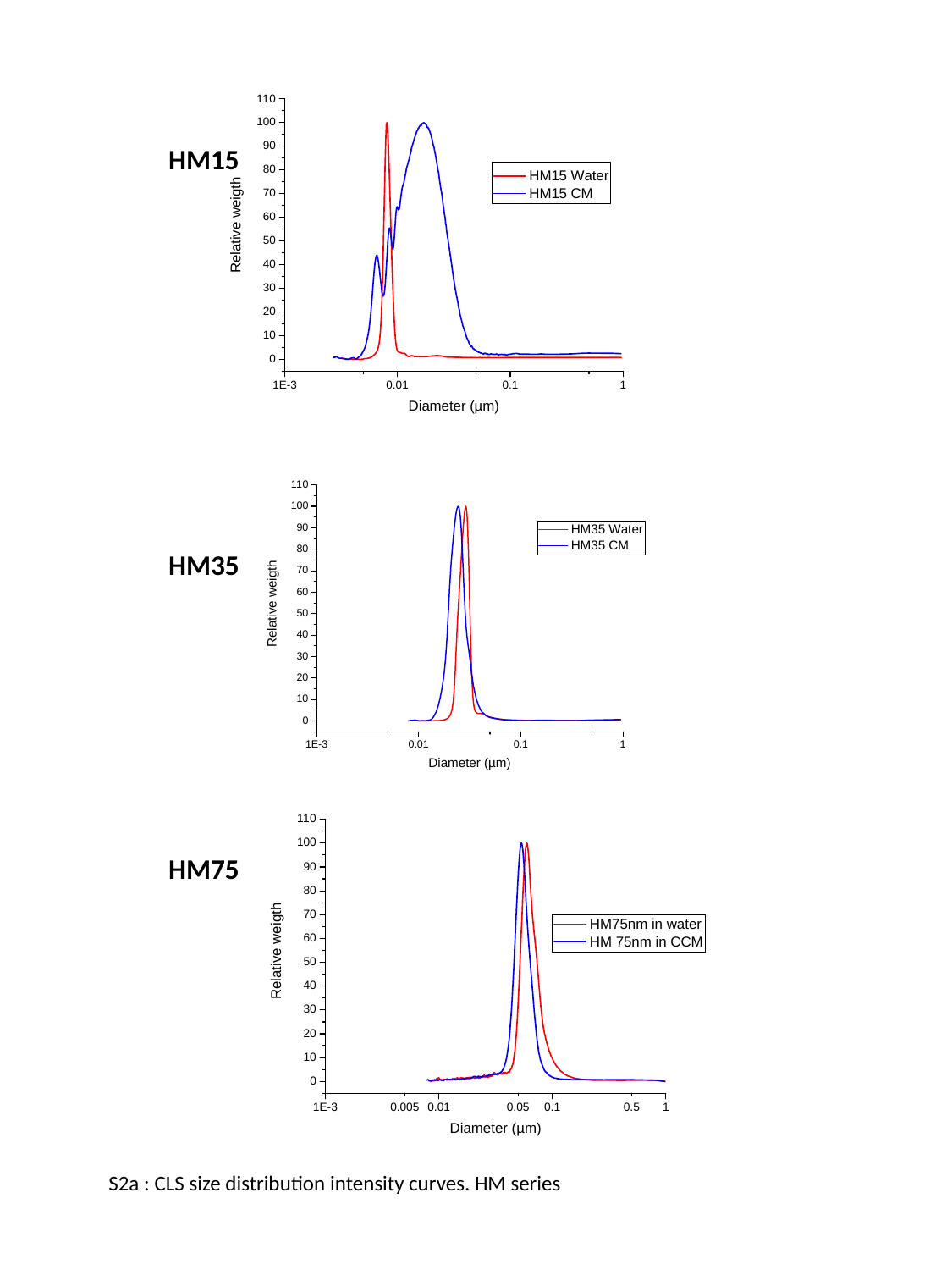

HM15
HM35
HM75
S2a : CLS size distribution intensity curves. HM series

## Slide 4
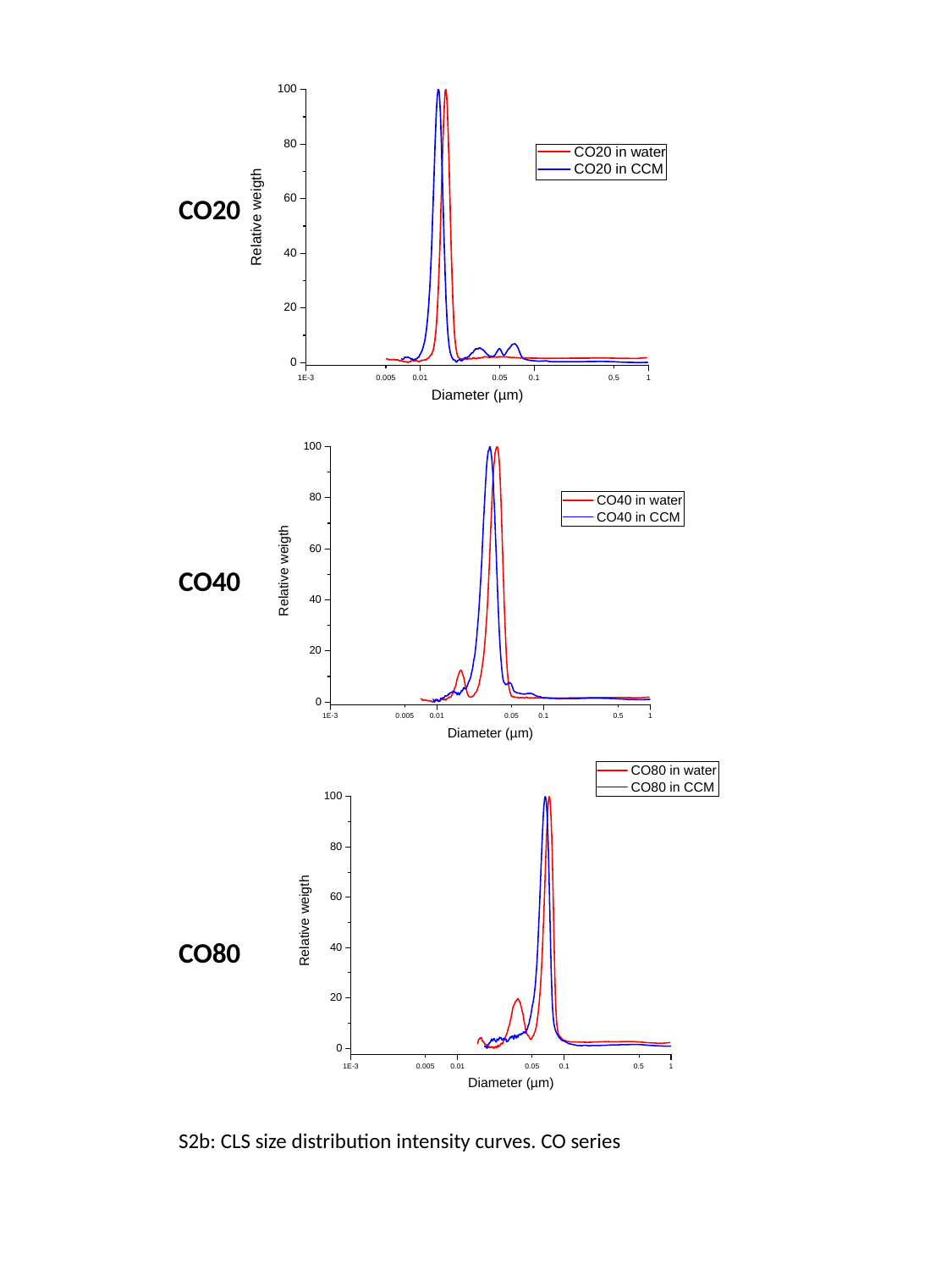

CO20
CO40
CO80
S2b: CLS size distribution intensity curves. CO series

## Slide 5
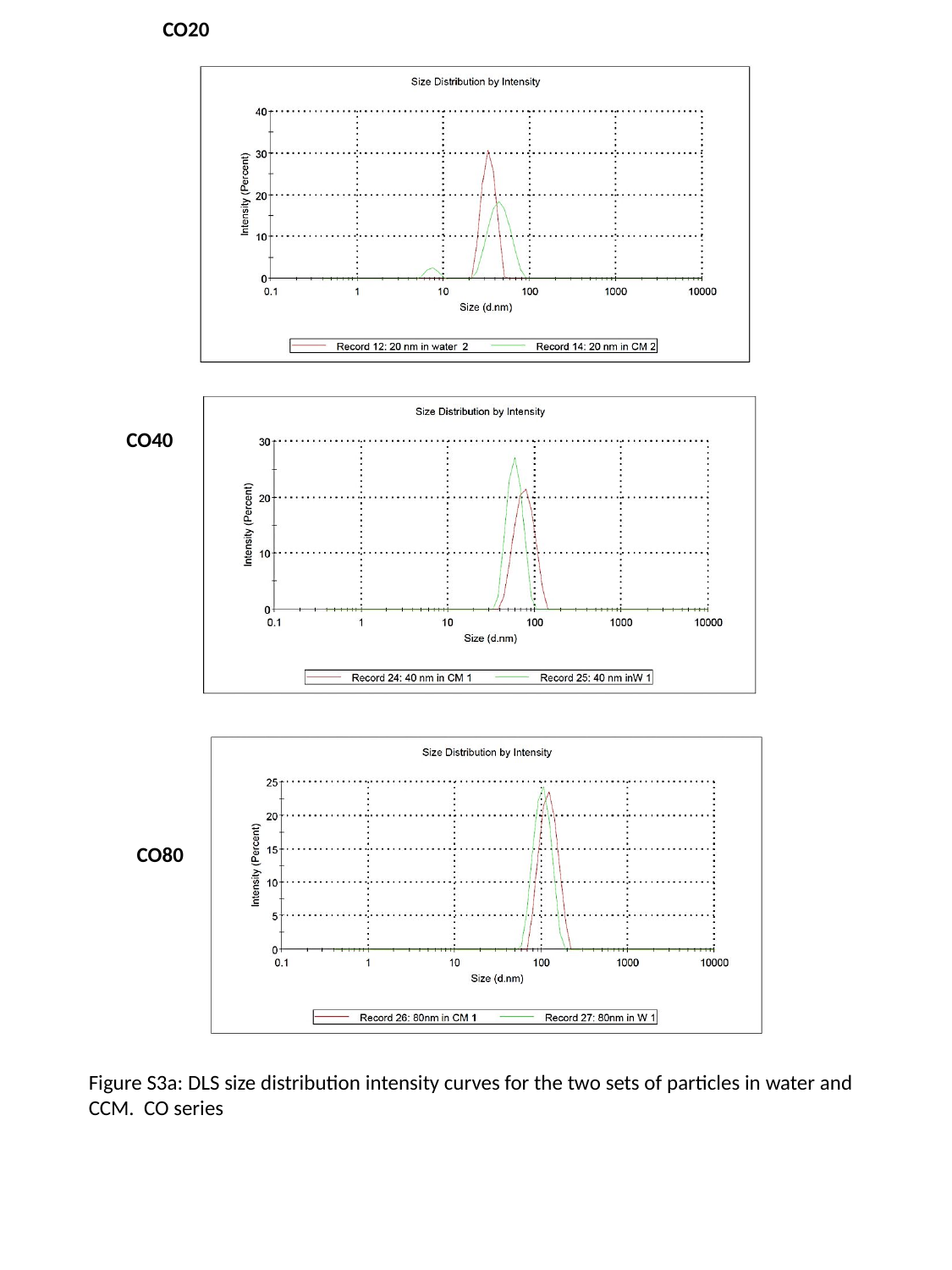

CO20
CO40
CO80
Figure S3a: DLS size distribution intensity curves for the two sets of particles in water and CCM. CO series

## Slide 6
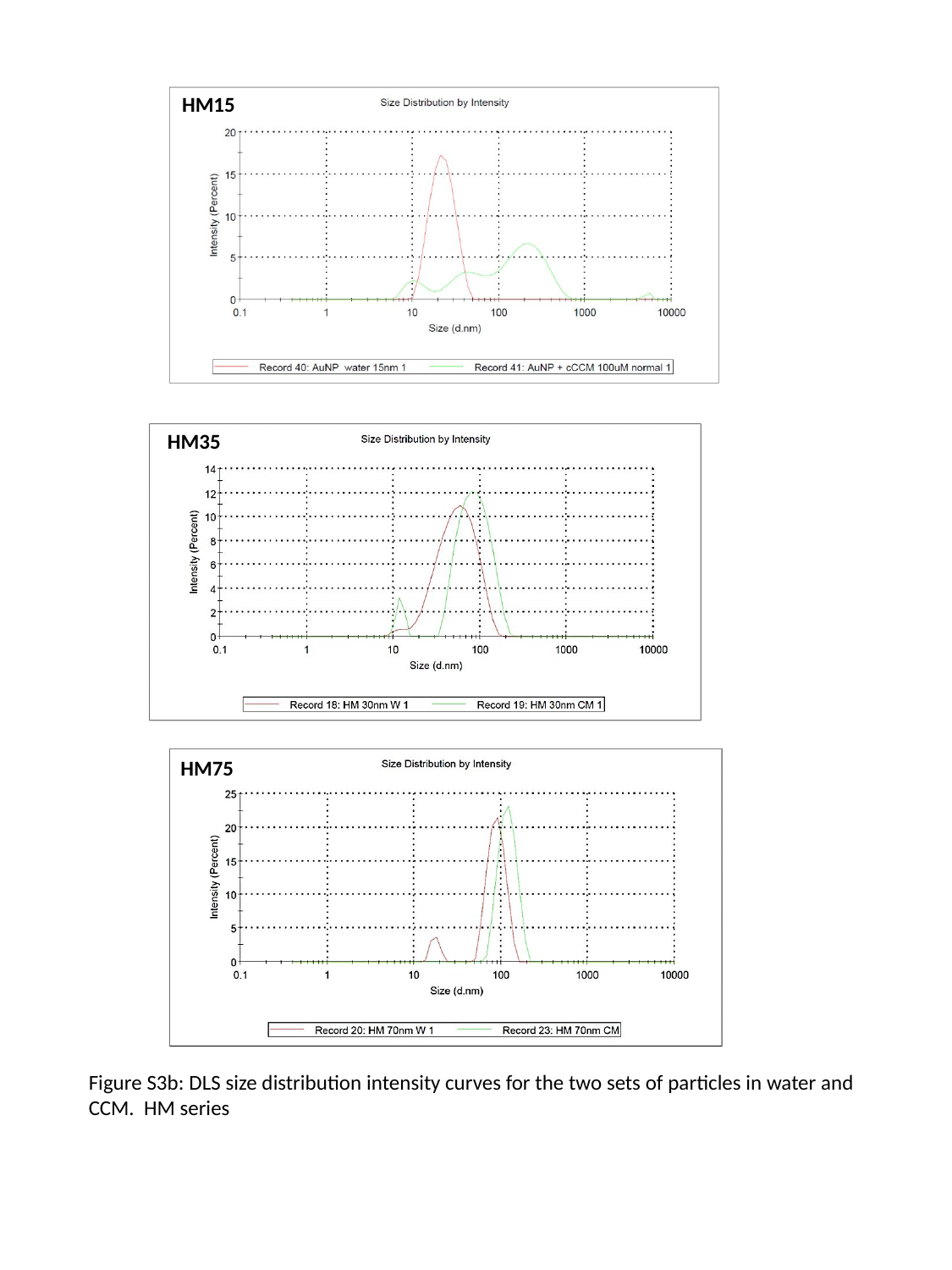

HM15
HM35
HM75
Figure S3b: DLS size distribution intensity curves for the two sets of particles in water and CCM. HM series

## Slide 7
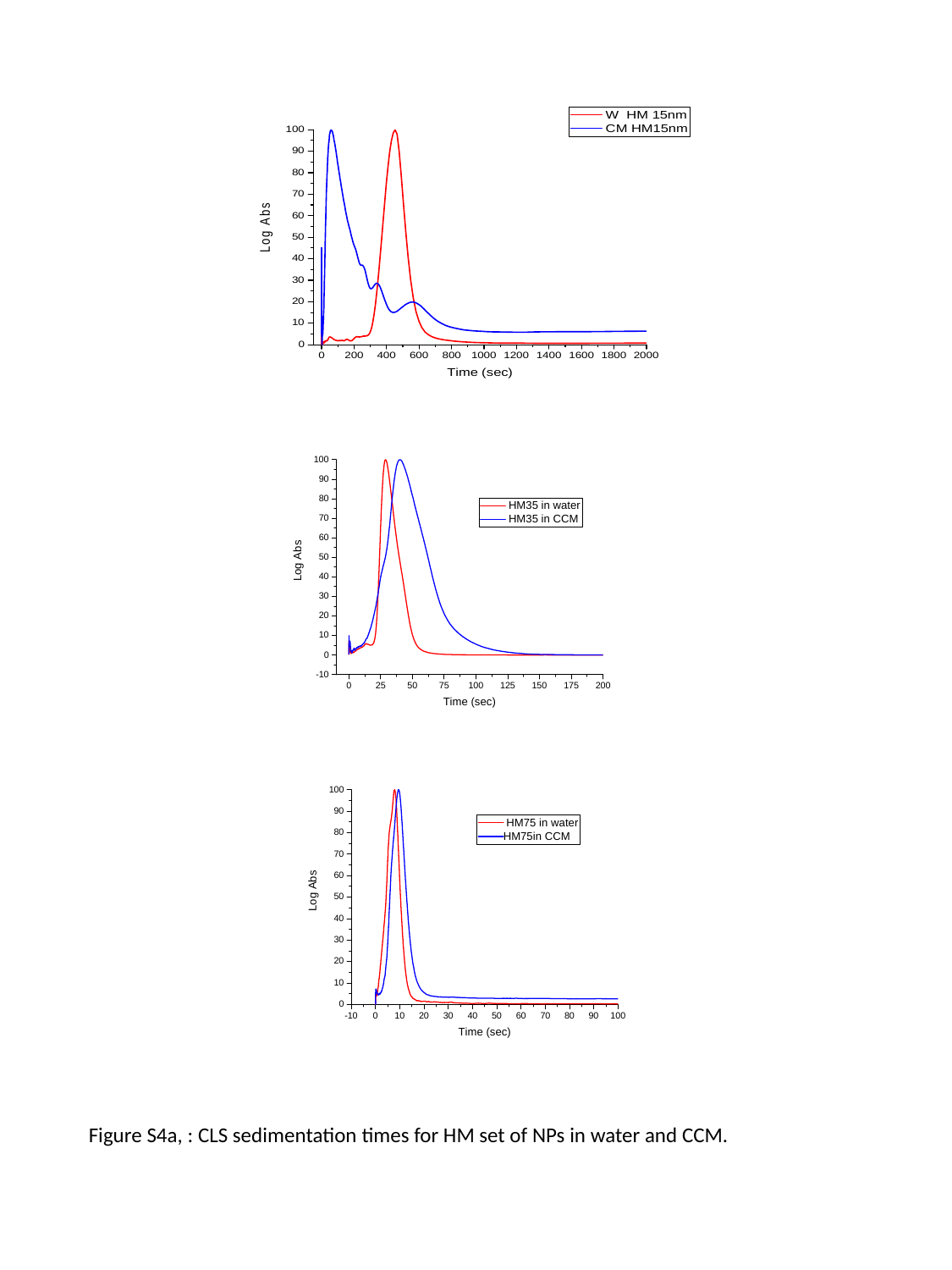

Figure S4a, : CLS sedimentation times for HM set of NPs in water and CCM.

## Slide 8
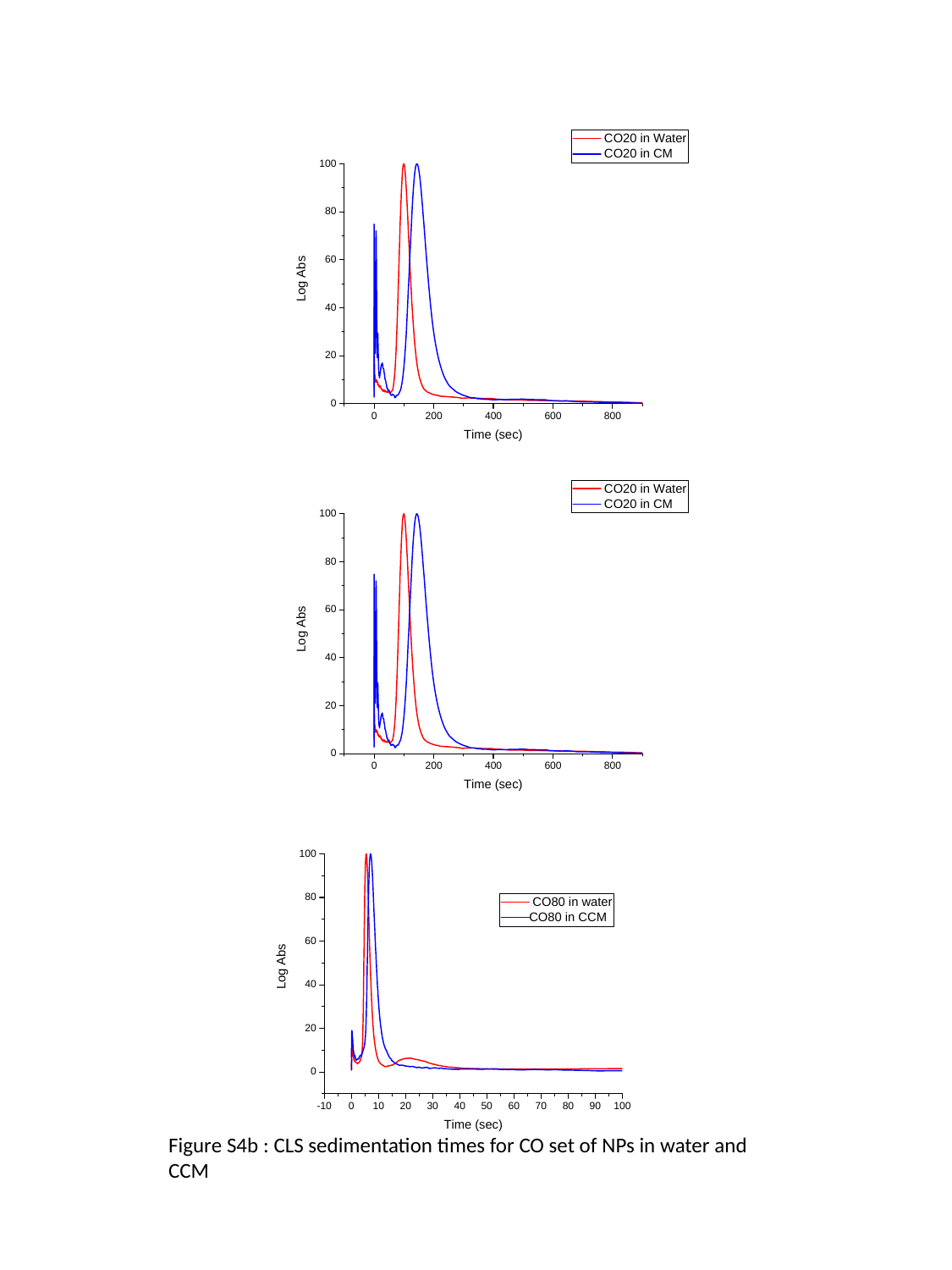

Figure S4b : CLS sedimentation times for CO set of NPs in water and CCM

## Slide 9
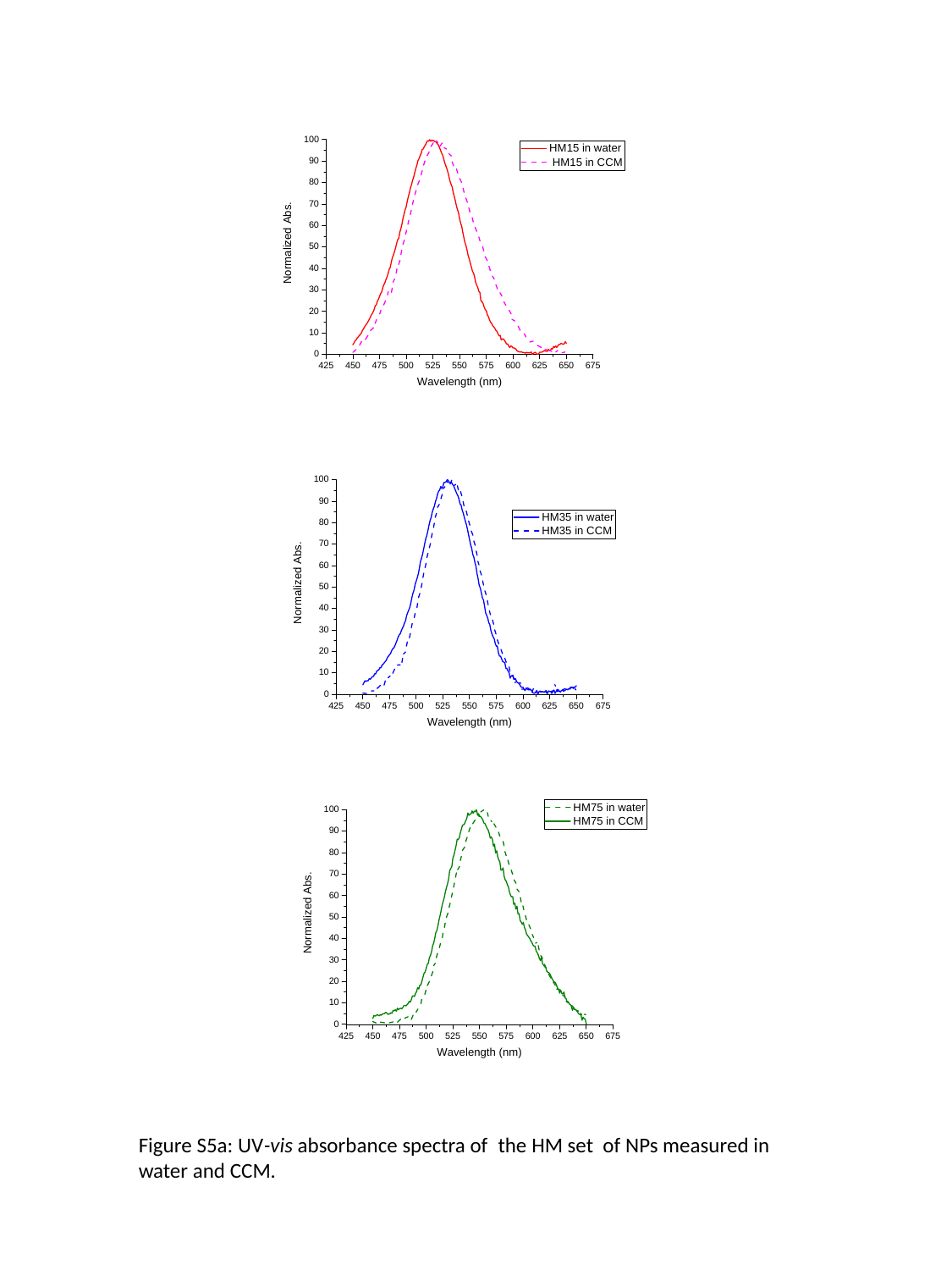

Figure S5a: UV-vis absorbance spectra of the HM set of NPs measured in water and CCM.

## Slide 10
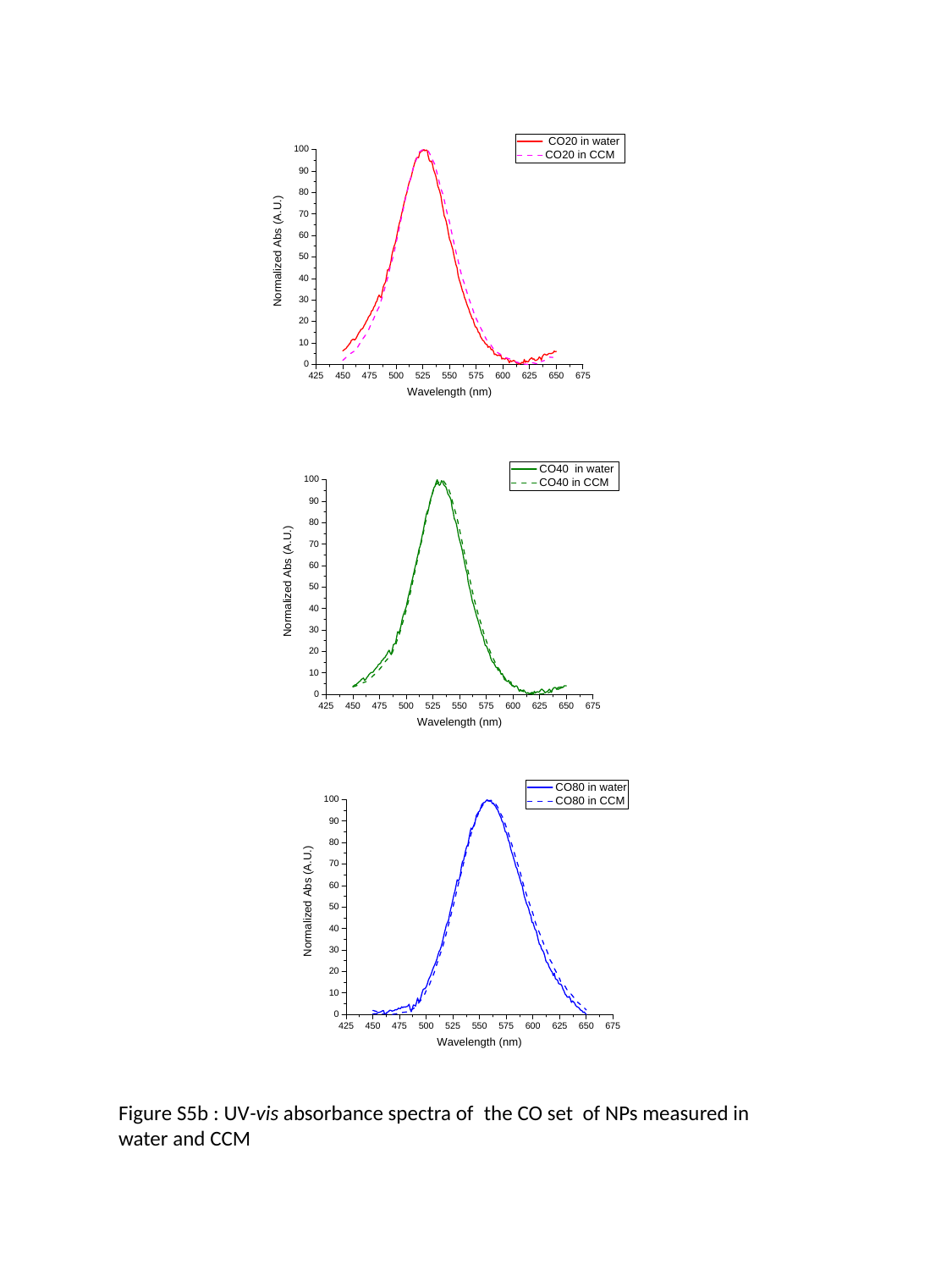

Figure S5b : UV-vis absorbance spectra of the CO set of NPs measured in water and CCM

## Slide 11
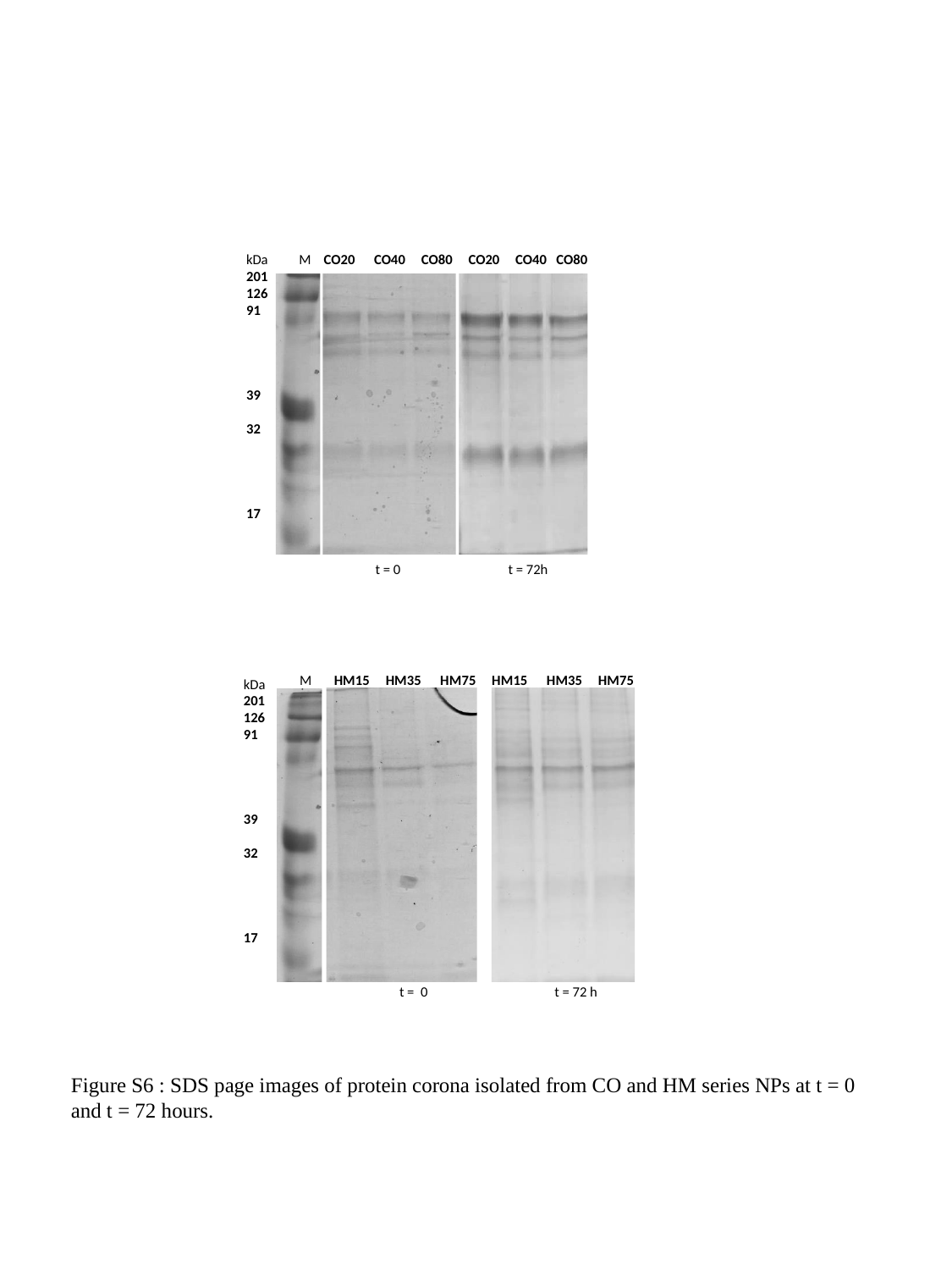

M CO20 CO40 CO80 CO20 CO40 CO80
kDa
201
126
91
39
32
17
t = 0
t = 72h
M HM15 HM35 HM75 HM15 HM35 HM75
kDa
201
126
91
39
32
17
t = 0
t = 72 h
Figure S6 : SDS page images of protein corona isolated from CO and HM series NPs at t = 0 and t = 72 hours.

## Slide 12
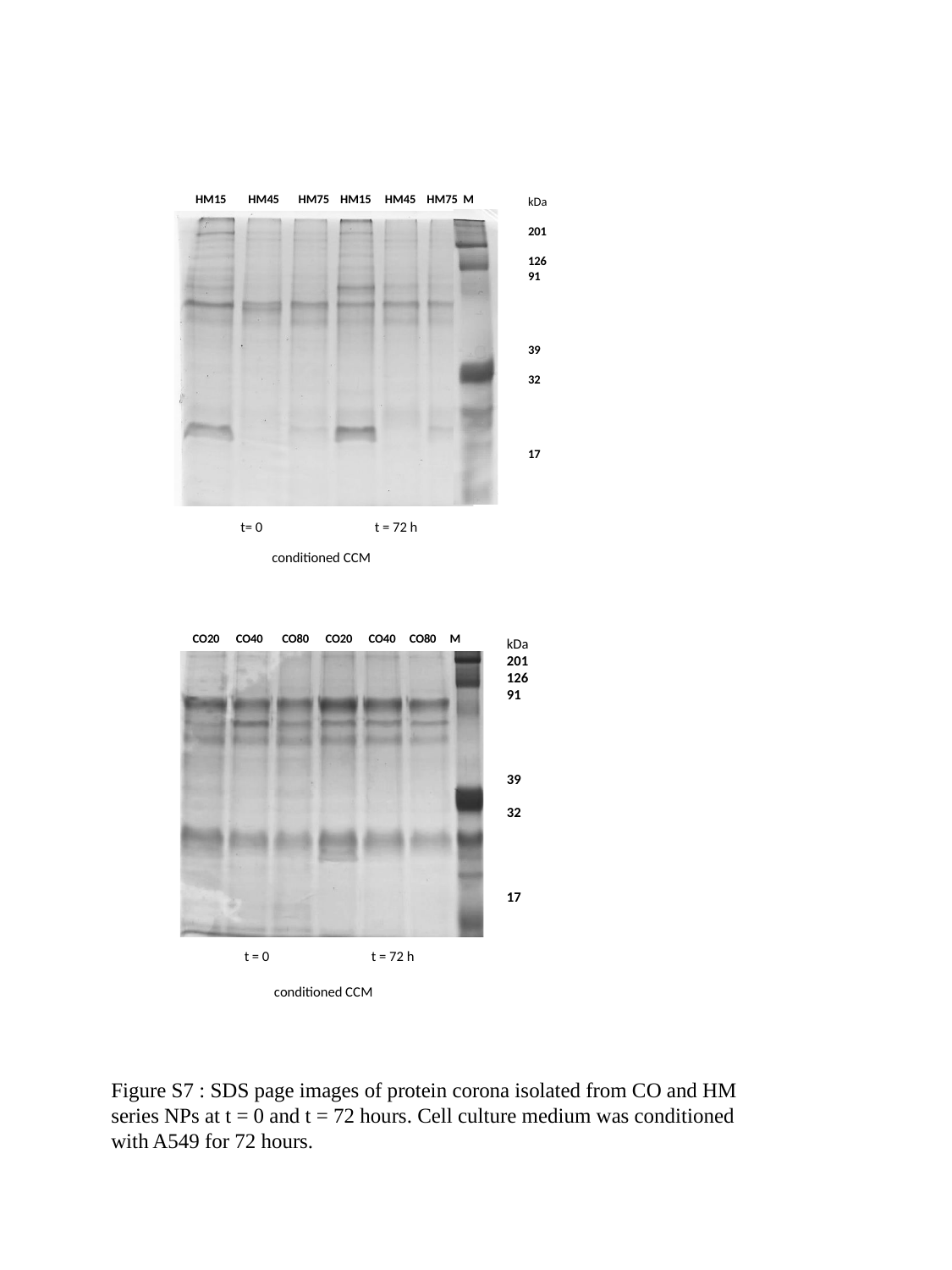

HM15 HM45 HM75 HM15 HM45 HM75 M
kDa
201
126
91
39
32
17
t= 0
t = 72 h
conditioned CCM
CO20 CO40 CO80 CO20 CO40 CO80 M
kDa
201
126
91
39
32
17
t = 0
t = 72 h
conditioned CCM
Figure S7 : SDS page images of protein corona isolated from CO and HM series NPs at t = 0 and t = 72 hours. Cell culture medium was conditioned with A549 for 72 hours.

## Slide 13
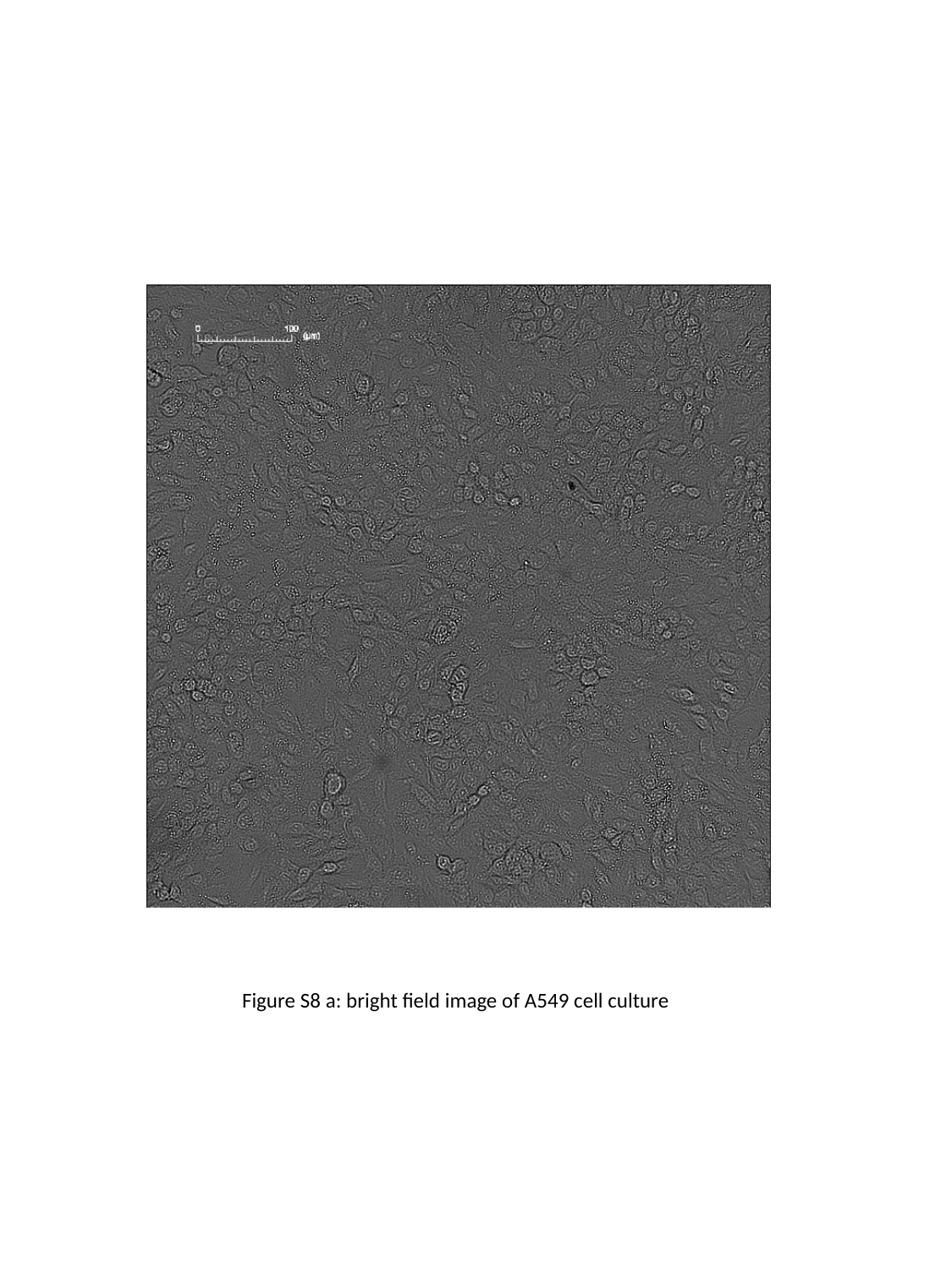

Figure S8 a: bright field image of A549 cell culture

## Slide 14
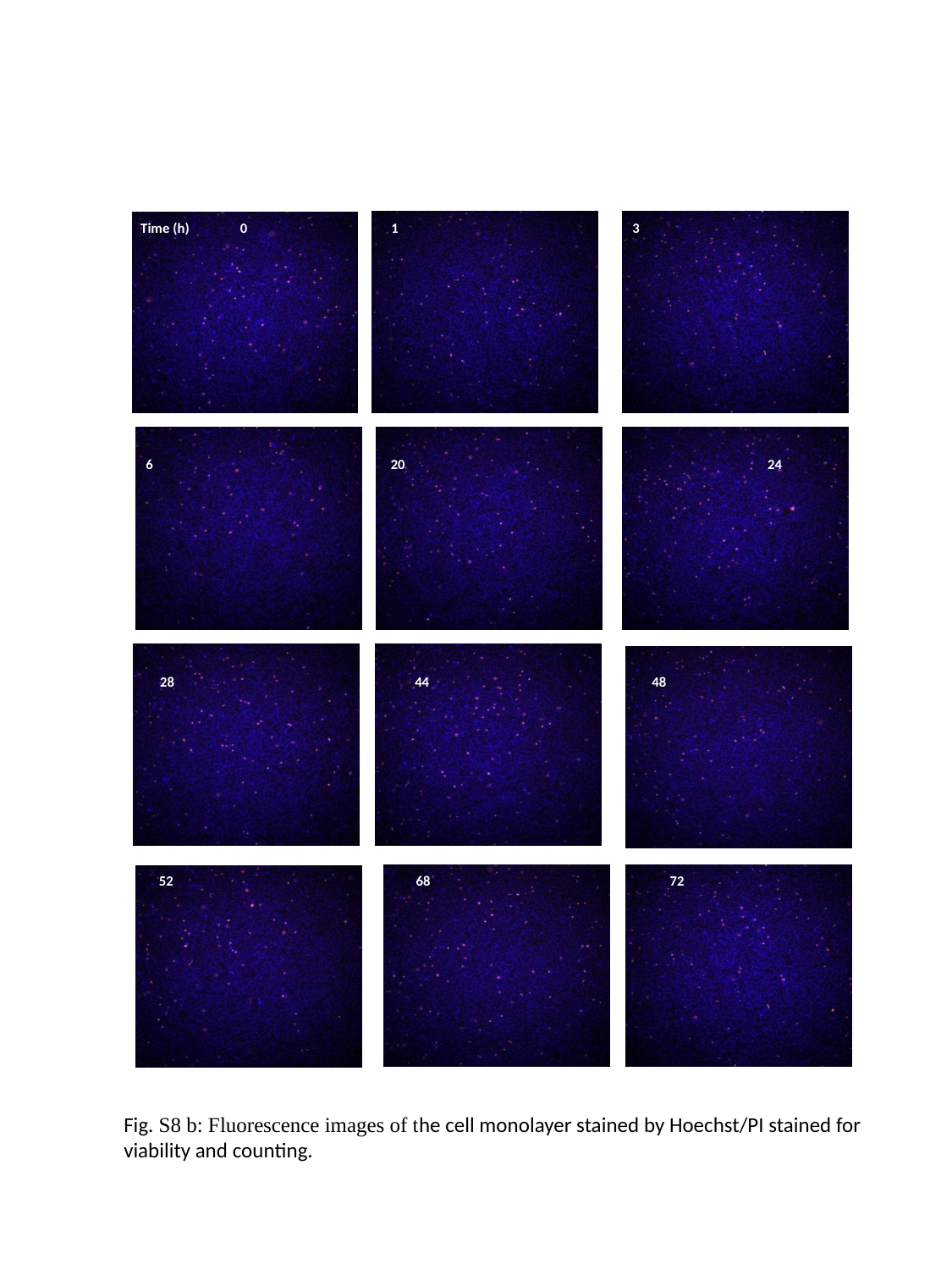

Time (h) 0	 1	 3
6 20	 24
28 44	 48
52		 68 	 	 72
Fig. S8 b: Fluorescence images of the cell monolayer stained by Hoechst/PI stained for viability and counting.

## Slide 15
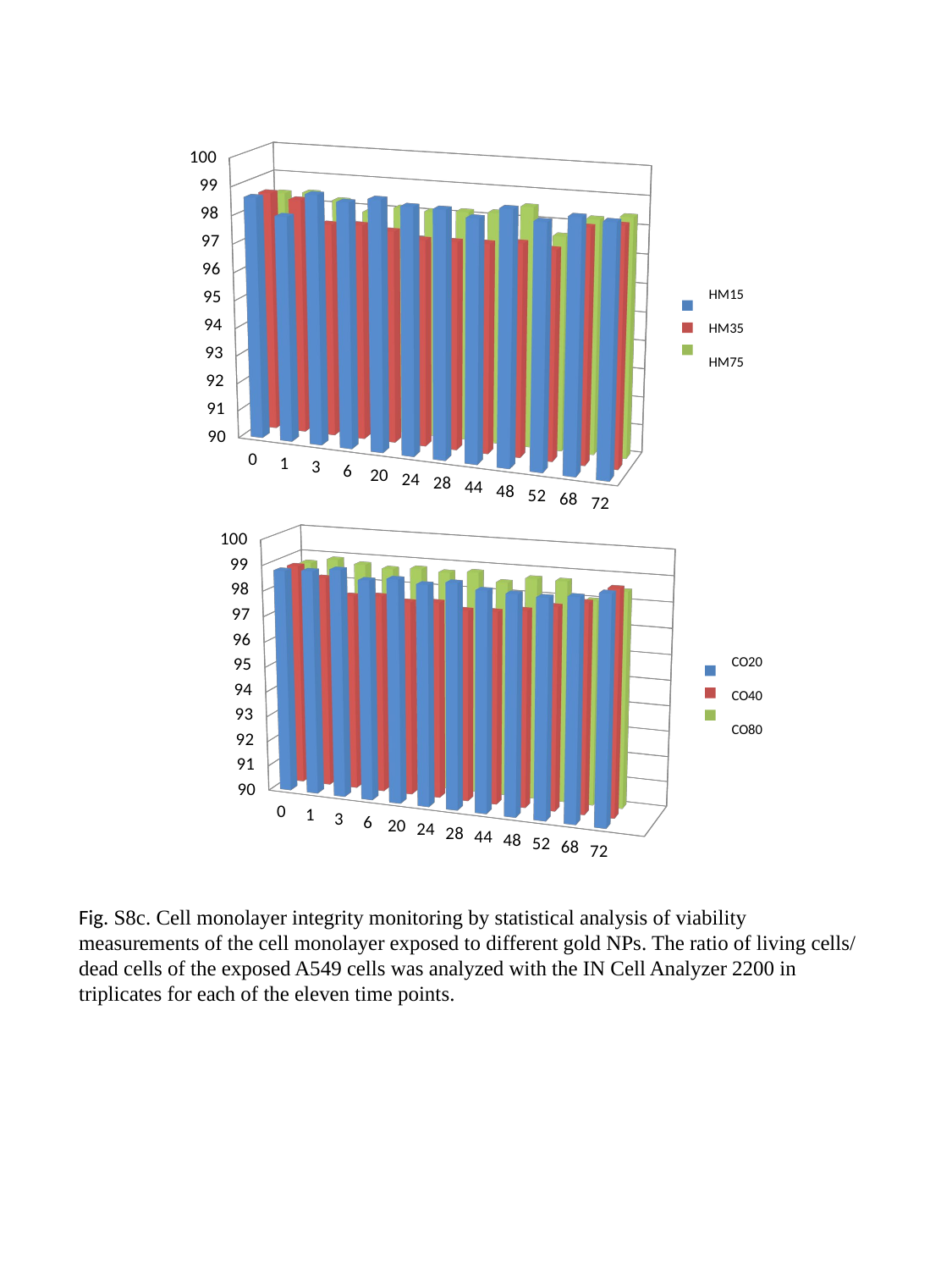

[unsupported chart]
HM15
HM35
HM75
[unsupported chart]
CO20
CO40
CO80
Fig. S8c. Cell monolayer integrity monitoring by statistical analysis of viability measurements of the cell monolayer exposed to different gold NPs. The ratio of living cells/ dead cells of the exposed A549 cells was analyzed with the IN Cell Analyzer 2200 in triplicates for each of the eleven time points.

## Slide 16
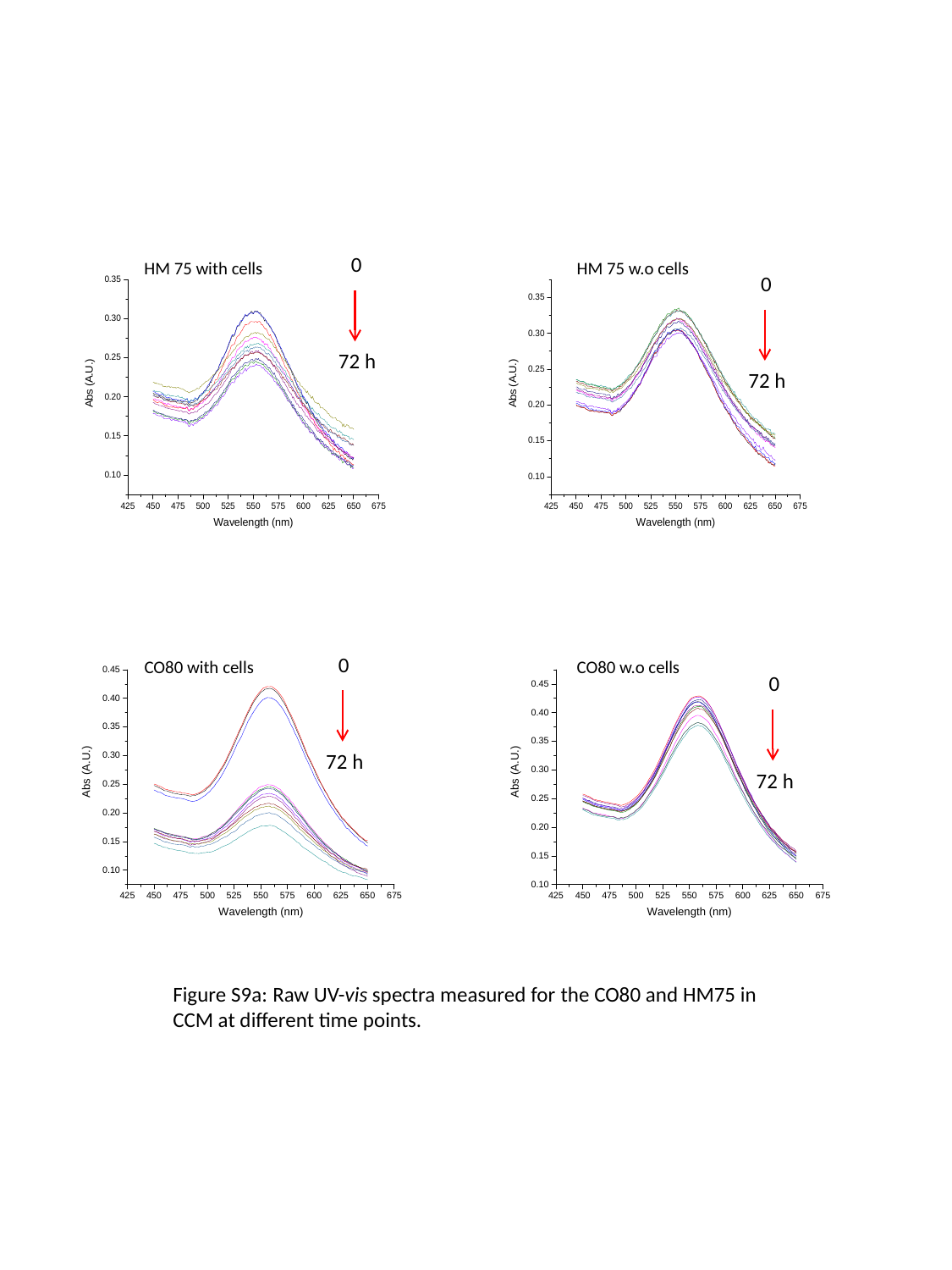

0
72 h
HM 75 with cells
HM 75 w.o cells
0
72 h
0
72 h
CO80 with cells
CO80 w.o cells
0
72 h
Figure S9a: Raw UV-vis spectra measured for the CO80 and HM75 in CCM at different time points.

## Slide 17
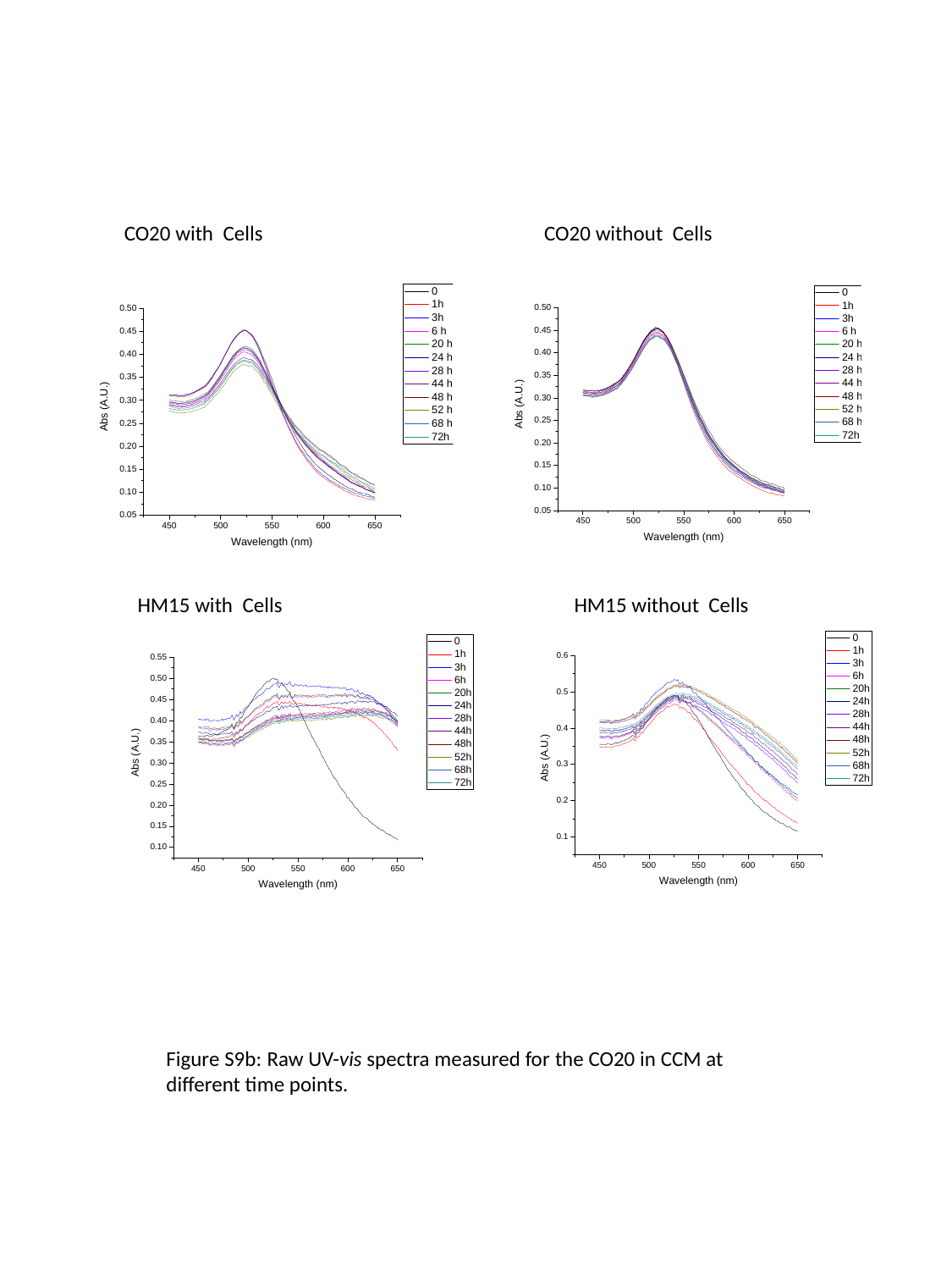

CO20 without Cells
CO20 with Cells
HM15 with Cells
HM15 without Cells
Figure S9b: Raw UV-vis spectra measured for the CO20 in CCM at different time points.

## Slide 18
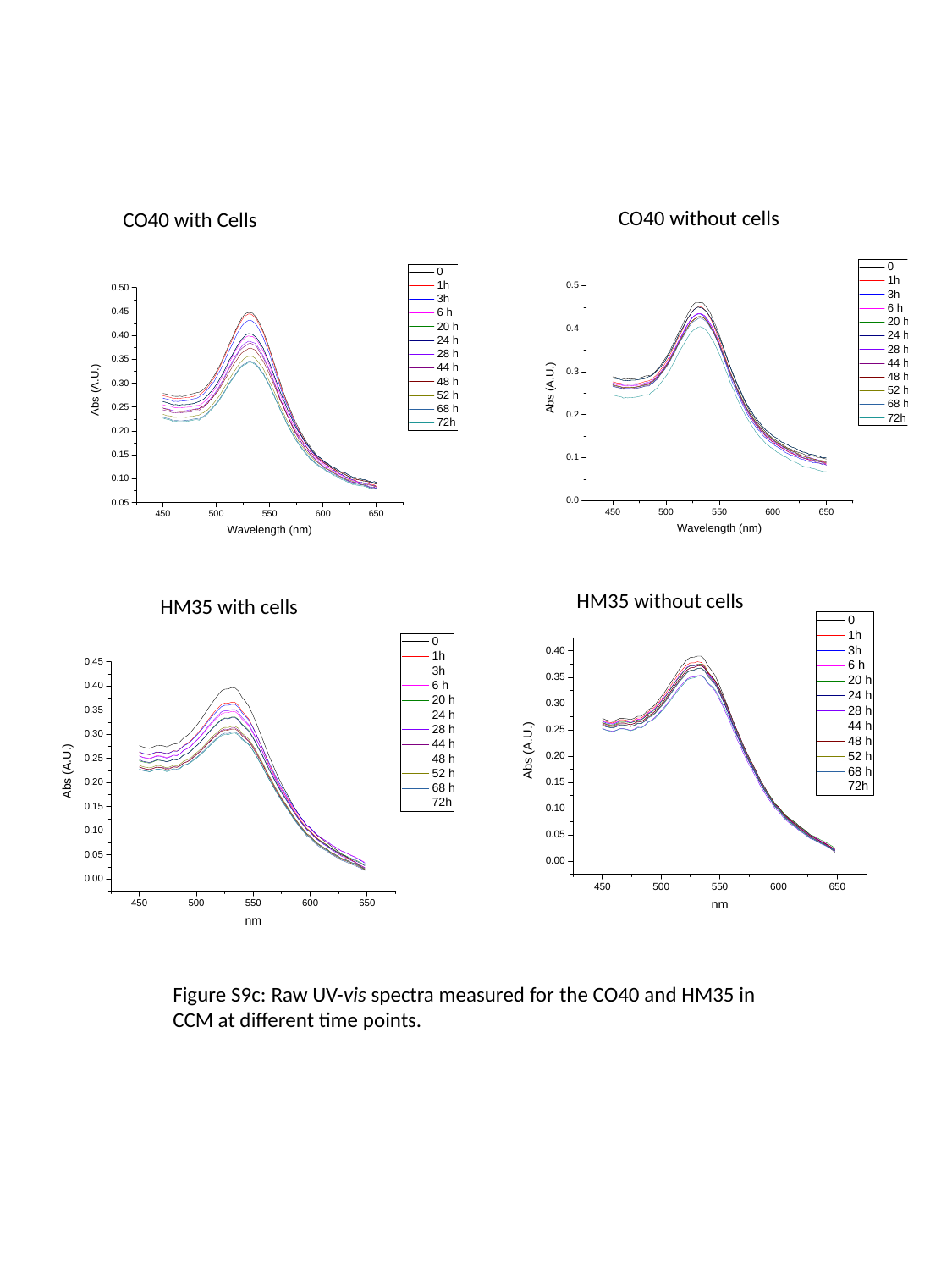

CO40 without cells
CO40 with Cells
HM35 without cells
HM35 with cells
Figure S9c: Raw UV-vis spectra measured for the CO40 and HM35 in CCM at different time points.

## Slide 19
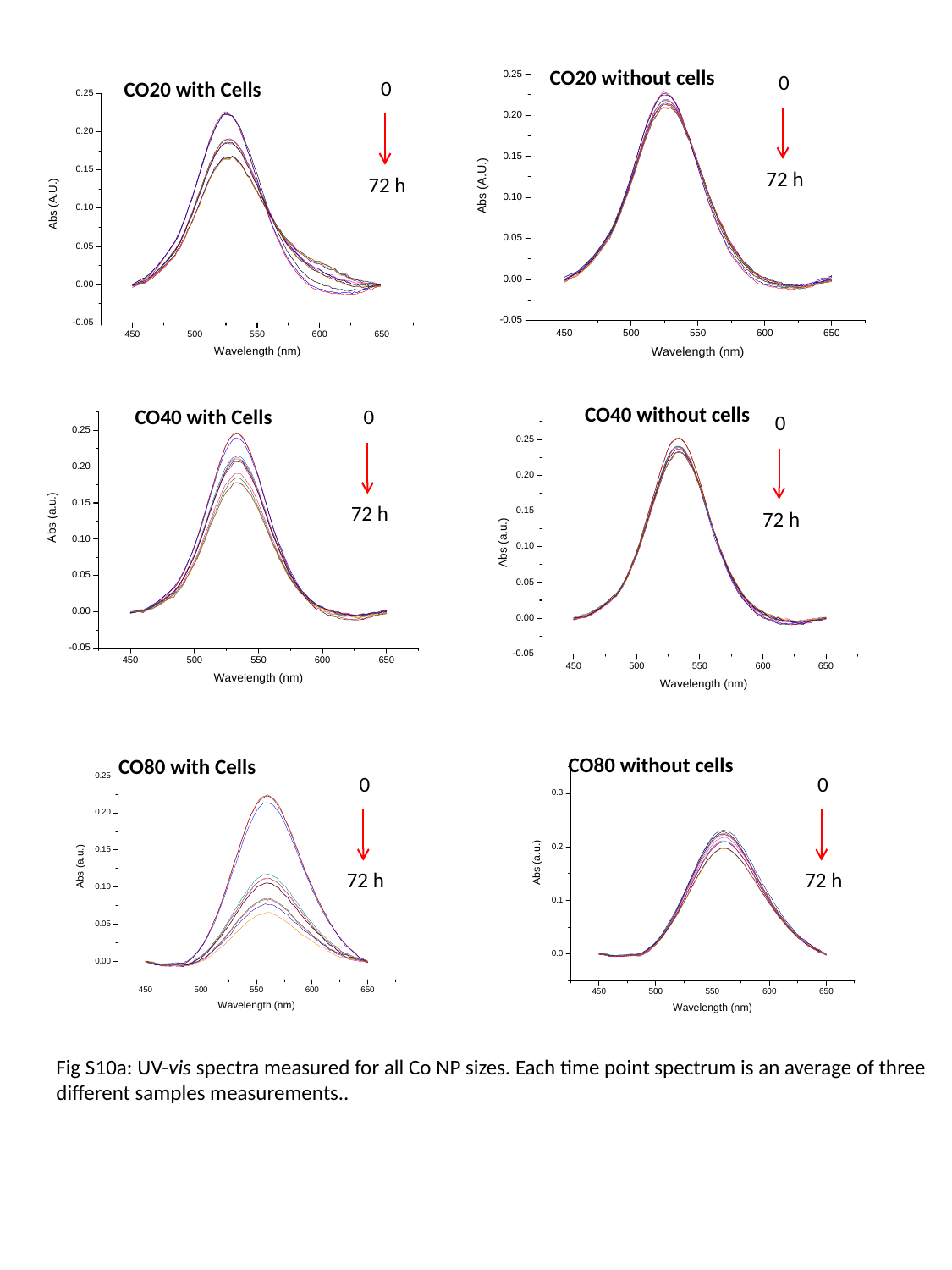

CO20 without cells
0
72 h
0
72 h
CO20 with Cells
CO40 without cells
CO40 with Cells
0
72 h
0
72 h
CO80 without cells
CO80 with Cells
0
72 h
0
72 h
Fig S10a: UV-vis spectra measured for all Co NP sizes. Each time point spectrum is an average of three different samples measurements..

## Slide 20
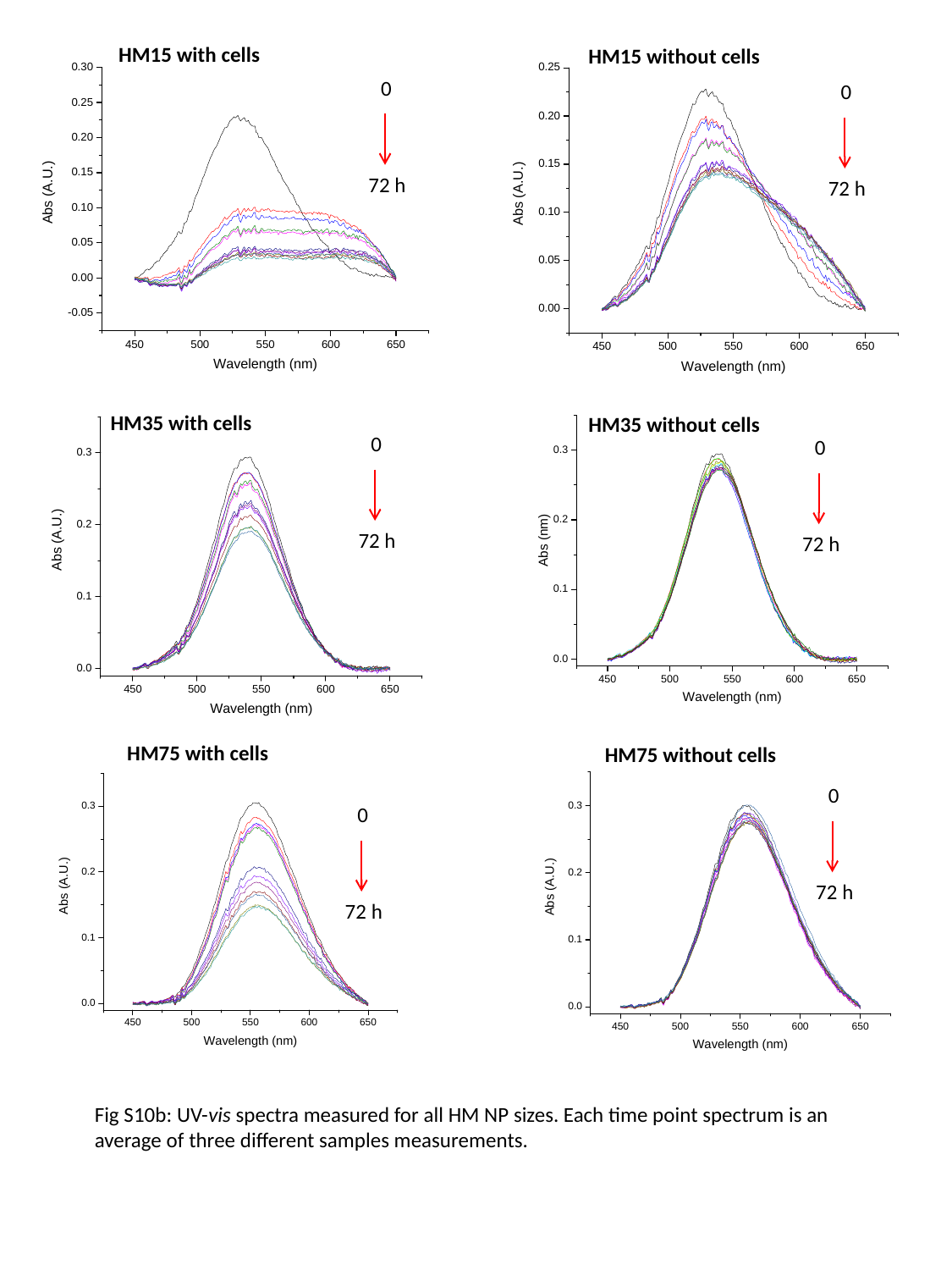

HM15 with cells
HM15 without cells
0
72 h
0
72 h
HM35 with cells
HM35 without cells
0
72 h
0
72 h
HM75 with cells
HM75 without cells
0
72 h
0
72 h
Fig S10b: UV-vis spectra measured for all HM NP sizes. Each time point spectrum is an average of three different samples measurements.

## Slide 21
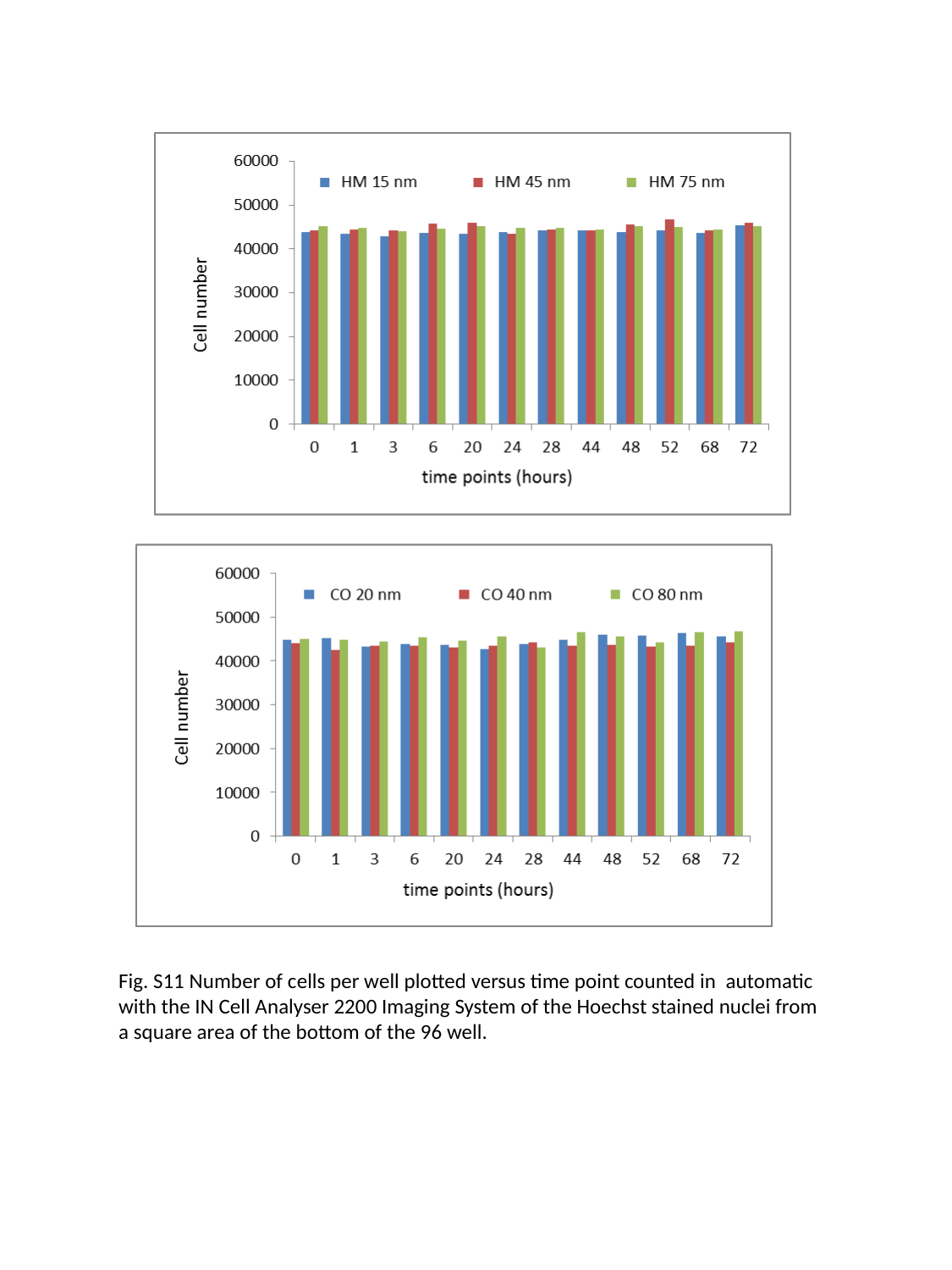

Fig. S11 Number of cells per well plotted versus time point counted in automatic with the IN Cell Analyser 2200 Imaging System of the Hoechst stained nuclei from a square area of the bottom of the 96 well.

## Slide 22
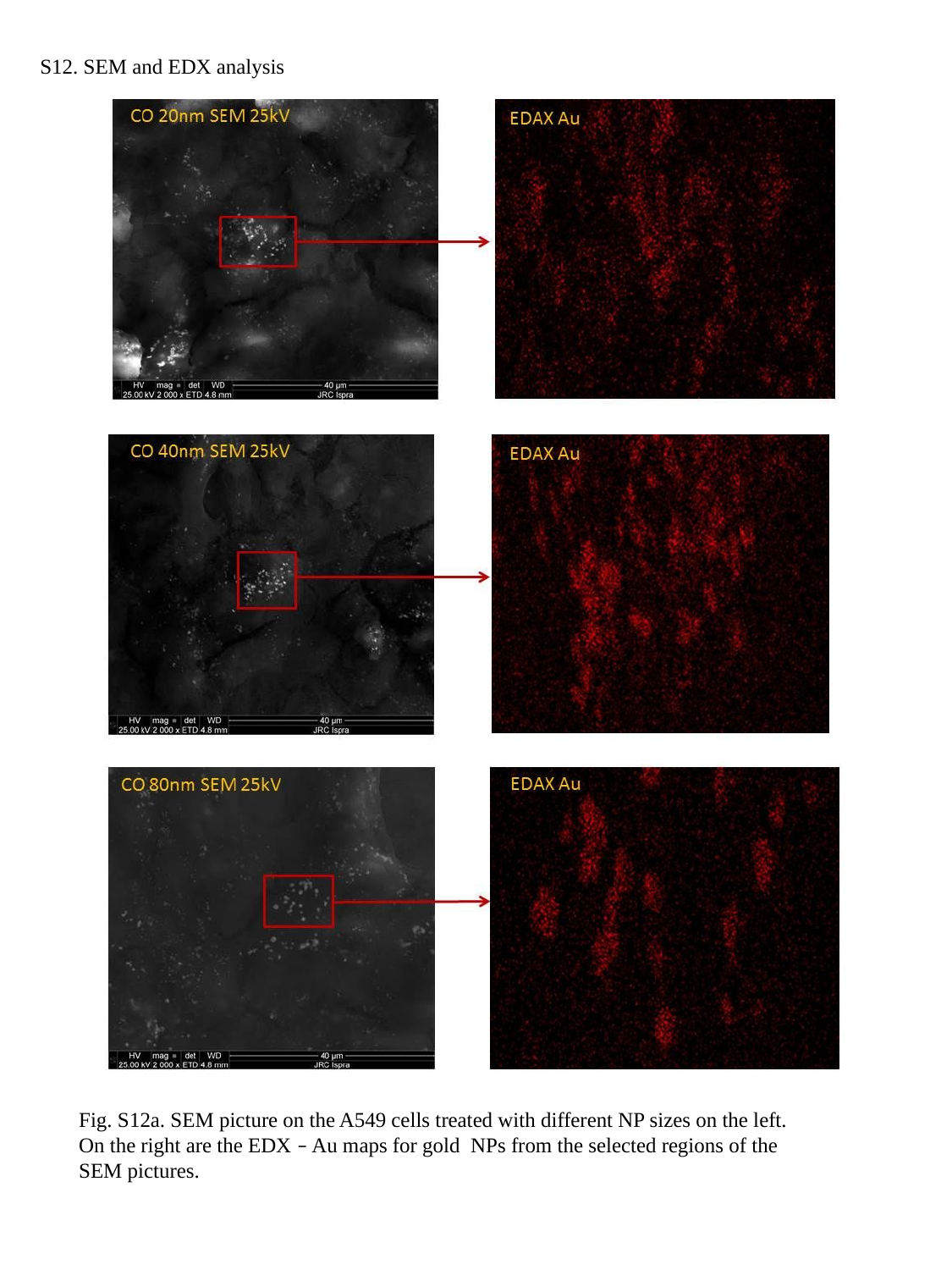

S12. SEM and EDX analysis
Fig. S12a. SEM picture on the A549 cells treated with different NP sizes on the left. On the right are the EDX – Au maps for gold NPs from the selected regions of the SEM pictures.

## Slide 23
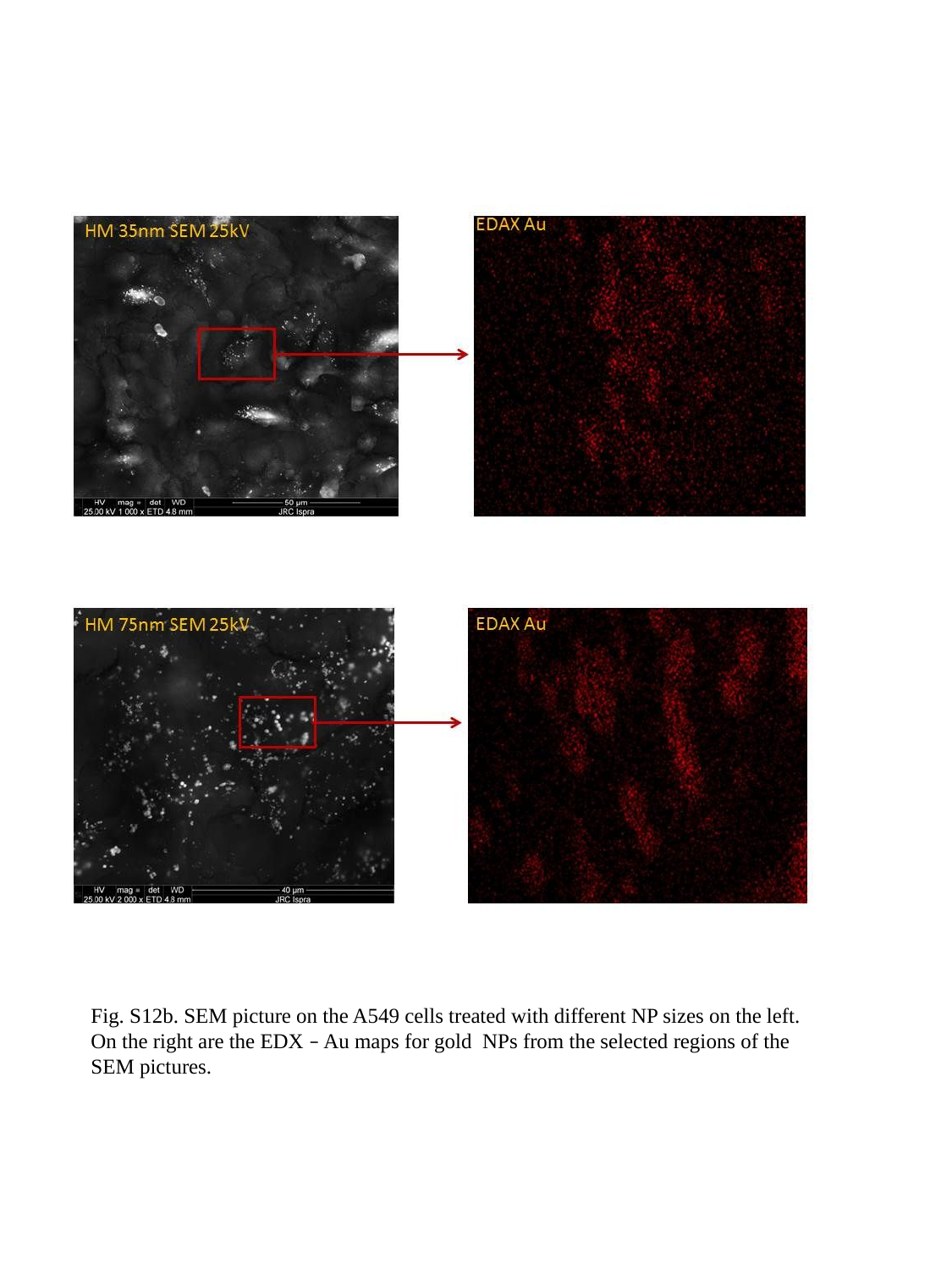

Fig. S12b. SEM picture on the A549 cells treated with different NP sizes on the left. On the right are the EDX – Au maps for gold NPs from the selected regions of the SEM pictures.
